# Histone lactylation-driven feedback loop modulates pyrimidine metabolism to promote oral carcinogenesis

**DOI:** 10.1038/s41419-026-08580-w

**Published:** 2026-03-19

**Authors:** Yanting Wang, Yanlin Geng, Yannan Chen, Haowen Zhang, Jingyu Liu, Yulin Song, Gang Wu, Tim Forouzanfar, Yuan Fan

**Affiliations:** 1 Department of Oral Mucosal Diseases, The Affiliated Stomatological Hospital of Nanjing Medical University, Nanjing, China; 2 State Key Laboratory Cultivation Base of Research, Prevention and Treatment for Oral Diseases, Nanjing, China; 3 Jiangsu Province Engineering Research Center of Stomatological Translational Medicine, Nanjing, China; 4 Stomatological College of Nanjing Medical University, Nanjing, China; 5 Department of Oral and Maxillofacial Surgery, Leiden University Medical Center (LUMC), Leiden, the Netherlands

**Keywords:** Oral cancer, Cancer genetics

## Abstract

Metabolic reprogramming and epigenetic alterations promote oral squamous cell carcinoma (OSCC). Lactate-dependent histone modification is a novel histone mark that connects the epigenetic process of lactylation to glycolytic metabolites. However, the role of histone lactylation in oral carcinogenesis remains poorly understood. In this study, the levels of histone lactylation in oral leukoplakia (OLK) and OSCC tissues were determined by immunohistochemistry. The involvement of histone lactylation in OSCC initiation was assessed by the inhibition of lactylation using glycolysis inhibitors or silencing lactate dehydrogenase A (LDHA), both in vitro and in vivo. CUT&Tag, scRNA-seq, ChIP-qPCR, and rescue experiments were conducted to explore the potential molecular mechanism of H3K18 lactylation (H3K18la) in OSCC tumorigenesis. Histone lactylation, particularly H3K18la levels were elevated in OLK and OSCC tissues. The inhibition of histone lactylation repressed the malignant phenotypes of OLK and OSCC cells in vitro. Glycolysis inhibitors blocked the formation of precancerous lesions and OSCC in the 4NQO-induced tongue carcinogenesis model. Mechanistically, H3K18la activated the transcription of thymidine kinase 1 (TK1) and increased TK1-mediated pyrimidine biosynthesis, resulting in oral carcinogenesis. TK1 downregulation inhibited the Wnt signaling pathway via RhoA. Moreover, the Wnt/β-catenin inhibitor XAV939 reduced lactate production and H3K18la levels. Here, we demonstrate that the glycolysis/H3K18la/TK1/β-catenin positive feedback loop exacerbates dysfunction in OSCC initiation. These findings reveal a novel link between epigenetic regulation and lactate-driven metabolic reprogramming, which may lead to the development of innovative lactylation treatment approaches for OSCC therapy.

## Introduction

Oral squamous cell carcinoma (OSCC) remains a leading cause of morbidity and mortality among individuals with head and neck squamous cell carcinoma (HNSCC) [1, 2]. Unfortunately, the 5-year survival rate for patients with OSCC has remained largely unchanged over the past several decades [3]. Although tobacco use, alcohol consumption, and genetic factors are considered primary risk factors for OSCC, the precise pathogenic mechanisms of OSCC remain mostly unknown [4]. Typical oral carcinogenesis begins with the normal epithelium that progresses to dysplasia and eventually to carcinoma. Oral leukoplakia (OLK), which is histologically characterized by features of dysplasia, is considered the most common oral precancerous lesion and has a malignant transformation rate ranging from 1.1% to 40.8% [5]. Thus, investigating the molecular mechanisms underlying OSCC development is crucial for identifying possible novel preventative and treatment targets.

Increased glycolysis and lactate accumulation are common features of many types of cancer [6]. As a byproduct of aerobic glycolysis, lactate promotes tumor cell growth, metastasis, and immunosuppression [7]. However, little is known about its nonmetabolic role in cancer cells. Lactylation, first identified in 2019, is an epigenetic modification that involves the modification of histone lysine residues to regulate gene transcription [8, 9]. The pioneering work demonstrated that lysine lactylation could be derived from lactate using isotopic sodium L-lactate (^13^C_3_) [8]. There are currently 28 known histone lactylation sites, such as histone H3 lysine 4 and histone H3 lysine 18 (H3K18) [10]. In non-small cell lung cancer, H3K18la directly activates the transcription of pore membrane protein 121, which enhances the nuclear transport of MYC and its binding to the CD274 promoter to induce PD-L1 expression [11]. The accumulation of lactate derived from colorectal cancer cells activates the transcription of NSUN2 through H3K18la, which is critical for capturing target RNAs [12]. Nonetheless, the role of histone lactylation in oral carcinogenesis remains largely unknown.

Thymidine kinase 1 (TK1) is a cytosolic enzyme that catalyzes the addition of a γ-phosphate group to thymidine and plays a key role in pyrimidine metabolism. TK1 contributes to DNA synthesis-related disorders, particularly various cancers, because of its enzymatic activity in deoxyribonucleotide metabolism [13]. Additionally, TK1 plays a significant role in the development of autoimmune diseases, myopathy, and HIV infection [14]. Despite these findings, the specific role of TK1 in OSCC tumorigenesis and its underlying molecular mechanisms remain largely unexplored.

In this study, we demonstrated that histone lactylation was driven by lactate accumulation in the OLK and OSCC microenvironments both in vivo and in vitro and was strongly associated with carcinogenesis. Mechanistically, increased glycolysis led to increases in lactate production and H3K18la levels, which promoted the transcription of TK1 and pyrimidine biosynthesis. Notably, TK1 activated lactate dehydrogenase A (LDHA), which further increased lactate production, and amplified histone lactylation via the RhoA/Wnt pathway, forming a glycolysis/H3K18la/TK1/β-catenin positive feedback loop. In summary, our findings reveal a novel role of histone lactylation, particularly H3K18la, in OSCC tumorigenicity and provide valuable insights into potential therapeutic targets for inhibiting oral carcinogenesis.

## Materials and methods

### Clinical specimen

Single-cell RNA sequencing (scRNA-seq) data of OLK tissues (*n* = 4) and primary OSCC tissues (*n* = 10) were obtained from the GSE181919 dataset. Clinical data and mRNA expression profiles were retrieved from The Cancer Genome Atlas (TCGA) database.

The tissue samples for validation were obtained from an independent cohort from the Department of Oral and Maxillofacial Surgery, including 30 normal tissue samples, 30 OLK samples and 30 OSCC samples (Table S1). This study was approved by the ethics committee of the Affiliated Hospital of Stomatology, Nanjing Medical University, and informed consent was obtained from all patients.

### Quality controlling, processing and clustering of scRNA-seq data

We read the gene expression matrix using the Seurat package. The data with low-quality cells expressing ≤ 500 genes or ≥ 8000 genes or the percentage of mitochondrial genes infinite or ≥ 15% were excluded, and the data with genes expressed in fewer than three cells were also removed. Batch effects of samples were corrected using “RunHarmony” in the “Harmony” R package. The FindClusters function was employed to perform cell-clustering and sub-clustering analyses with appropriate resolutions. The identified cell clusters and sub-clusters were displayed using the uniform manifold approximation and projection (UMAP). The cell clusters were annotated on the basis of highly expressed genes, distinctly expressed genes, and published canonical cellular markers.

### Functional analysis

Differentially expressed genes (DEGs) were analyzed using the DAVID database (https://david.ncifcrf.gov/) for Reactome and Gene Ontology (GO) pathway enrichment. The pathways were selected on the basis of ranking by count.

### Cell culture

The human OSCC cell lines (HN4 and HN6) and the human oral keratinocyte cell line (HOK) were provided by the Chinese Academy of Sciences. The human OSCC cell lines (HSC3, CAL27 and UPCI-SCC-154) were obtained from the American Type Culture Collection (VA, USA). Human dysplastic oral keratinocyte (DOK) cells were generously provided by Prof. Cheng Bin (Sun Yat-sen University, China). The human OSCC cell lines (HSC3, HN4, HN6, and CAL27) and HOK were cultured as previously described [15]. UPCI-SCC-154 was grown in Eagle’s Minimum Essential Medium, enriched with 10% FBS and 2 mM L-glutamine [16].

### Measurement of lactate levels

As directed by the manufacturer, a Lactate Colorimetric/Fluorometric Assay Kit (BioVision, CA, USA) was used to quantify the levels of lactate. In brief, buffer was used to lyse the cell pellet or tissue, and the supernatant was collected by centrifugation. The assay solution was then mixed with the supernatant. A spectrophotometric plate reader was used to measure the absorbance at 570 nm. The results were normalized based on the protein levels.

### Immunohistochemistry (IHC)

IHC was performed as previously described [17]. The antibodies used are listed in Table S2. Two pathologists independently evaluated the immunohistochemical staining results. The percentage of positively stained cells was multiplied by the staining intensity to determine the staining index (SI). On a scale of 0–4, the proportion of positively stained cells was evaluated as follows: 1 (<10%), 2 (10–35%), 3 (35–70%), or 4 (>70%). Staining intensity was scored as 0 (negative), 1 (weak), 2 (moderate), or 3 (strong). P53 positivity was defined as ≥ 10% of tumor cells with nuclear staining as described previously [18, 19].

### Western blotting assay

RIPA buffer supplemented with a protease inhibitor cocktail (CWBIO, China) was used to extract total protein. In accordance with the manufacturer’s instructions, nuclear protein was extracted using an NE-PER™ Nuclear Cytoplasmic Extraction Reagent kit (Thermo Fisher, Massachusetts, USA). Western blotting assay was performed as previously described [20]. The antibodies used are listed in Table S2.

### Cell viability and colony formation assays

For the cell viability assay, 1000 cells were seeded into each well of 96-well plates. Cell Counting Kit-8 (CCK-8; Telenbiotech, Guangzhou, China) was then added to each well at specific time points (1, 2, 3, 4, and 5 days), and the cells were incubated for 2 h according to the manufacturer’s instructions. A microplate reader was used to measure the absorbance at 450 nm.

For the colony formation assay, 1000 cells were seeded into each well of the six-well plates and cultured for one to two weeks. After fixation with paraformaldehyde solution, the cells were stained with crystal violet.

### Flow cytometry analyses

For cell cycle analysis, cells were synchronized by serum starvation in serum-free medium for 24 h, followed by incubation in medium containing 10% FBS for another 24 h. In accordance with the directions provided by the manufacturer of the cell cycle kit (Multi Sciences, Hangzhou, China), the cells were then resuspended with 1 mL DNA staining solution and 10 µL permeabilization solution and incubated for 30 min at room temperature. The cell cycle distribution was analyzed using a flow cytometer (Beckman Coulter, CA, USA).

For the apoptosis assay, an Annexin V-FITC/PI apoptosis kit (Multi Sciences, Hangzhou, China) was used. Briefly, cells were cultivated with FBS-free culture medium. After 24 h of starvation, the cells were collected and stained with PI and FITC for 5 min at room temperature. The apoptotic cells were determined by flow cytometry.

### Transwell assay

Transwell chambers (BD, NJ, USA) were used for cell migration and invasion assays. Briefly, for the migration assay, DOK (8 × 10^4^) or CAL27 (3 × 10^5^) cells were seeded into the upper chambers of the Transwell plates in serum-free medium. For the invasion assay, DOK (1.1 × 10^5^) or CAL27 (3.3 × 10^5^) cells were plated into the upper chambers of Transwell plates precoated with Matrigel. Medium supplemented with 10% FBS was placed in the lower chambers. After incubation for 24 h or 48 h, the cells on the opposite side of the inserts were stained with crystal violet solution and observed under an inverted microscope (Leica, Wetzlar, Germany).

### RNA interference and plasmid transfection

Small interfering RNAs (siRNAs) and negative control (NC) were produced by GenePharma (Shanghai, China). Lipofectamine RNAiMAX transfection reagent (Invitrogen, CA, USA) was used for transient transfection according to the manufacturer’s guidelines. The cells were harvested 48 h after transfection with 50 nmol·L^-1^ siRNA oligonucleotides. The sequences of the siRNAs used are listed in Table S3.

The short hairpin RNA (shRNA) targeting TK1 (sh-TK1), TK1 overexpression plasmid (TK1-OE), and their corresponding negative controls were obtained from GeneChem (Shanghai, China). Transfection was performed using Lipofectamine 2000 (Invitrogen, CA, USA) as previously described [21]. Knockdown or overexpression of TK1 was confirmed using Western blotting analysis.

### Extracellular acidification rate (ECAR) assay

The ECAR was measured using the Seahorse XF96 Pro analyzer (Agilent Technologies, CA, USA). The cells were stimulated with glucose, oligomycin, and 2-DG to evaluate the changes in the ECAR. At the time of measurement, the number of cells in each plate was used to standardize the results.

### Animal experiments

All animal experiments were conducted in accordance with the protocols approved by the Institutional Animal Care and Use Committee of Nanjing Medical University. Thirty-six female C57BL/6 mice (6–8 weeks old) were acquired from GemPharmatech (Nanjing, China). The mice received 100 μg/mL 4-nitroquinoline-1-oxide (4NQO; Sigma-Aldrich, Missouri, USA) in their drinking water for 16 weeks. At week 17, the mice were randomly assigned to three groups (*n* = 12 for each group) and intraperitoneally injected twice per week with 2-DG (500 mg/kg), oxamate (500 mg/kg), or vehicle control. At the end of week 20, the mice were euthanized.

Ten female BALB/c nude mice (5 weeks old) were randomly divided into two groups (five individuals per group). CAL27 cells with NC or TK1 knockdown (2 × 10^6^ cells) were injected into the dorsal flank of the mice. Following a 4-day examination, the tumor volumes were determined every 3 days for 3 weeks. Finally, the mice were sacrificed, and the tumors were dissected and weighed.

### CUT&Tag assay

The CUT&Tag assay was performed by Jiayin Biotechnology Ltd. (Shanghai, China). Briefly, the cells were bound to concanavalin A-coated beads, resuspended in antibody buffer, and sequentially incubated with primary and secondary antibodies against H3K18la. The pA-Tn5 transposase and samples were incubated at the same time. To construct the library, transposon activation and tagmentation were followed by DNA isolation, amplification, and purification. Post-PCR clean-up was conducted using XP beads (Beckman Coulter, CA, USA). The size distribution of libraries was assessed by Agilent 4200 TapeStation, and libraries were mixed to achieve equal representation. Sequencing was performed in the Illumina NovaSeq 6000 using 150 bp paired-end following the manufacturer’s instructions.

### RNA sequencing

For RNA extraction, the cells were treated with TRIzol reagent according to the manufacturer’s protocol. Subsequently, sequencing libraries were generated using the NEBNext^®^ Ultra^TM^ RNA Library Prep Kit for Illumina^®^ (NEB, USA). Finally, the libraries were sequenced on the Illumina NovaSeq platform.

### Chromatin immunoprecipitation quantitative PCR (ChIP-qPCR)

To investigate the interaction between H3K18la and the promoter region of TK1, a SimpleChIP^®^ Plus Enzymatic Chromatin IP Kit (CST, Massachusetts, USA) was used following the manufacturer’s procedure. The primers used for ChIP-qPCR are listed in Table S3.

### Real-time RT-PCR

Total RNA was extracted from cells with an RNA extraction reagent kit (YEASEN, Shanghai, China). Complementary DNA was synthesized via reverse transcription with a PrimeScript^TM^ RT reagent kit (Takara, Kyoto, Japan). Real-time RT-PCR was performed using a SYBR^®^ Premix Ex Taq^TM^ kit (Takara, Kyoto, Japan). The 2^–ΔΔCt^ method was used to determine relative expression. The primers used are listed in Table S3.

### Quantitative LC-MS/MS analysis for intracellular nucleotides measurement

Cells were seeded in 100 mm dishes and subsequently collected. Nucleotides were extracted using 100 µl ice-cold methanol/acetonitrile (50% V/V) containing stable isotopically labeled internal standards. This was followed by three freeze-thaw cycles (freeze for 30 seconds in liquid nitrogen and then thaw by sonication for 30 min at 4 °C). The proteins were precipitated by centrifugation at 13,000 rpm at 4 °C for 15 min, and the supernatants were further diluted with 400 µL of water. A Vanquish UHPLC coupled to a TSQ Altis triple quadrupole mass spectrometer (Thermo Fisher, Massachusetts, USA) was utilized to analyze the clear metabolite extract.

### Rho GTPase activity detection

The levels of GTP-bound RhoA were measured using a Rho Activation Assay Kit (Cell Biolabs, CA, USA) in accordance with the manufacturer’s guidelines. After being starved for 24 h in serum-free medium and stimulated for 2 h with 10% FBS, the cells were harvested using lysis buffer. Rhotekin RBD agarose beads were added to the cell lysates, which were subsequently incubated for 1 h at 4 °C. GTP-RhoA expression was detected via western blotting assays.

### Statistical analysis

The data were processed and analyzed using R 4.3.1, SPSS 25 and GraphPad Prism 8.0. The results from at least three independent experiments are presented as mean ± SD. *P* < 0.05 was regarded as statistically significant on the basis of two-sided tests. Student’s *t* test was used to compare continuous variables. χ^2^ test or Fisher’s exact test was used to compare categorical variables. Receiver operating characteristic curves were used to determine the optimal cutoff values for high and low gene expression. Survival curves were plotted using the Kaplan-Meier method, and differences in survival rates were estimated using the log-rank test. Spearman’s rank correlation coefficients were calculated to assess the bivariate correlations between variables.

## Results

### Histone lactylation levels increase during OSCC initiation

**Fig. 1:**
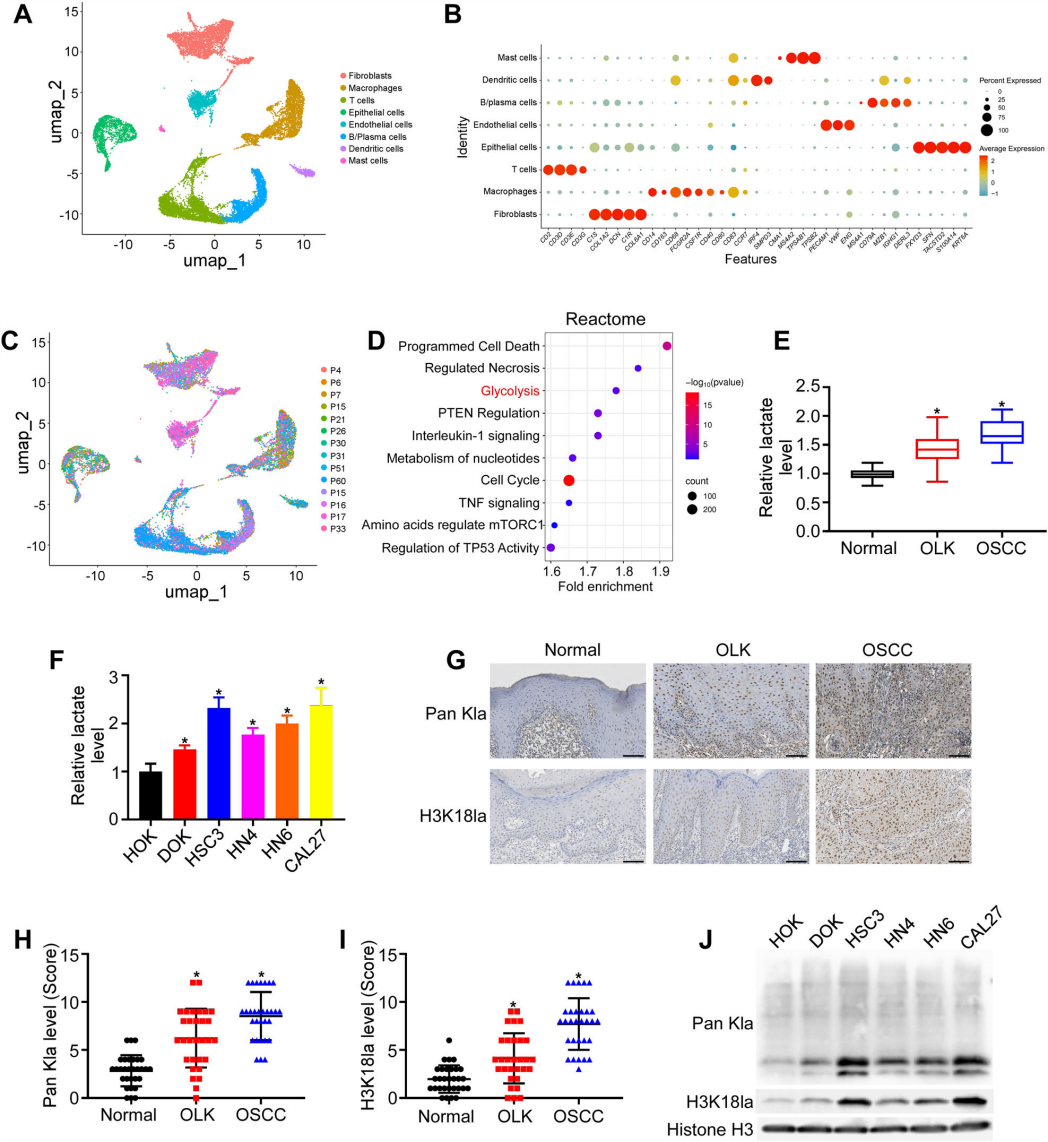
Histone lactylation increases during OSCC initiation. **A** UMAP plot of the clustering results for eight major cell types from OLK and OSCC tissues. **B** Marker genes of each cluster. **C** UMAP plot showing the cell distribution across all 14 samples. **D** Reactome pathway analysis of DEGs with gradually increased expression between epithelial cells from OLK and primary OSCC. **E** Lactate content in normal (*n* = 30), OLK (*n* = 30), and OSCC (*n* = 30) tissues. **F** Lactate content in HOK, DOK, and OSCC cells (HSC3, HN4, HN6, and CAL27). The representative images (×200) (**G**) and quantification analysis of Pan Kla (**H**) and H3K18la (**I**) protein levels in normal (*n* = 30), OLK (*n* = 30), and OSCC (*n* = 30) tissues were assessed by immunohistochemical staining. Scale bar: 100 μm. (**J**) Pan Kla and H3K18la levels in human HOK, DOK, and OSCC cells (HSC3, HN4, HN6, and CAL27) were analyzed by western blotting assays. Error bars, mean ± SD; ^*^P < 0.05.

The transition from OLK to OSCC marks the tipping point for tumorigenesis [22]. To elucidate the mechanisms underlying OSCC initiation, scRNA-seq profiles from 4 patients with OLK and 10 patients with primary OSCC (GSE181919) were analyzed. On the basis of the molecular markers of each cell type, a total of 16,822 cells were clustered into distinct types, including fibroblasts, macrophages, T cells, epithelial cells, endothelial cells, B/plasma cells, dendritic cells, and mast cells (Fig. 1A–C). The epithelial cells from OLK and primary OSCC were then selected, and Reactome pathway analysis of DEGs with increased expression trend were enriched in pathways related to metabolism, especially glycolysis (Fig. 1D).

Lactate is an important product of glycolysis and has been identified as an epigenetic modulator of gene regulation and tumor growth [23, 24]. The lactate content was examined using our own clinical cohort. Notably, the lactate content was higher in OSCC (*n* = 30) tissues than in OLK (*n* = 30) and normal mucosa (*n* = 30) tissues, with the lowest level detected in normal tissues (Fig. 1E). Similarly, lactate production was higher in OSCC cells (HSC3, HN4, HN6, and CAL27) than in HOK and DOK cells, with the lowest production observed in HOK cells (Fig. 1F). Histone lactylation is a novel posttranslational modification (PTM) in which lactate is used as a substrate. We examined lactylation levels in OLK and OSCC tissues. Immunohistochemical staining of normal (*n* = 30), OLK (*n* = 30), and OSCC (*n* = 30) samples revealed significantly elevated global lactylation levels in OLK and OSCC tissues compared with normal tissues (Fig. 1G, H). Given that most biological processes depend on histone modifications and that H3K18la has been shown to regulate various biological processes, such as oncogenesis [10], we focused on its level and function. Moreover, H3K18la levels were consistently increased in OLK and OSCC tissues (Fig. 1G, I). We further evaluated the lactylation levels in cell lines using western blotting assays and observed elevated levels of pan-lysine lactylation (Pan Kla) and H3K18la in DOK and OSCC cells (Fig. 1J). Together, these results suggest that histone lactylation may serve as a biomarker during oral carcinogenesis.

### Inhibition of histone lactylation suppresses OLK and OSCC cell progression

**Fig. 2:**
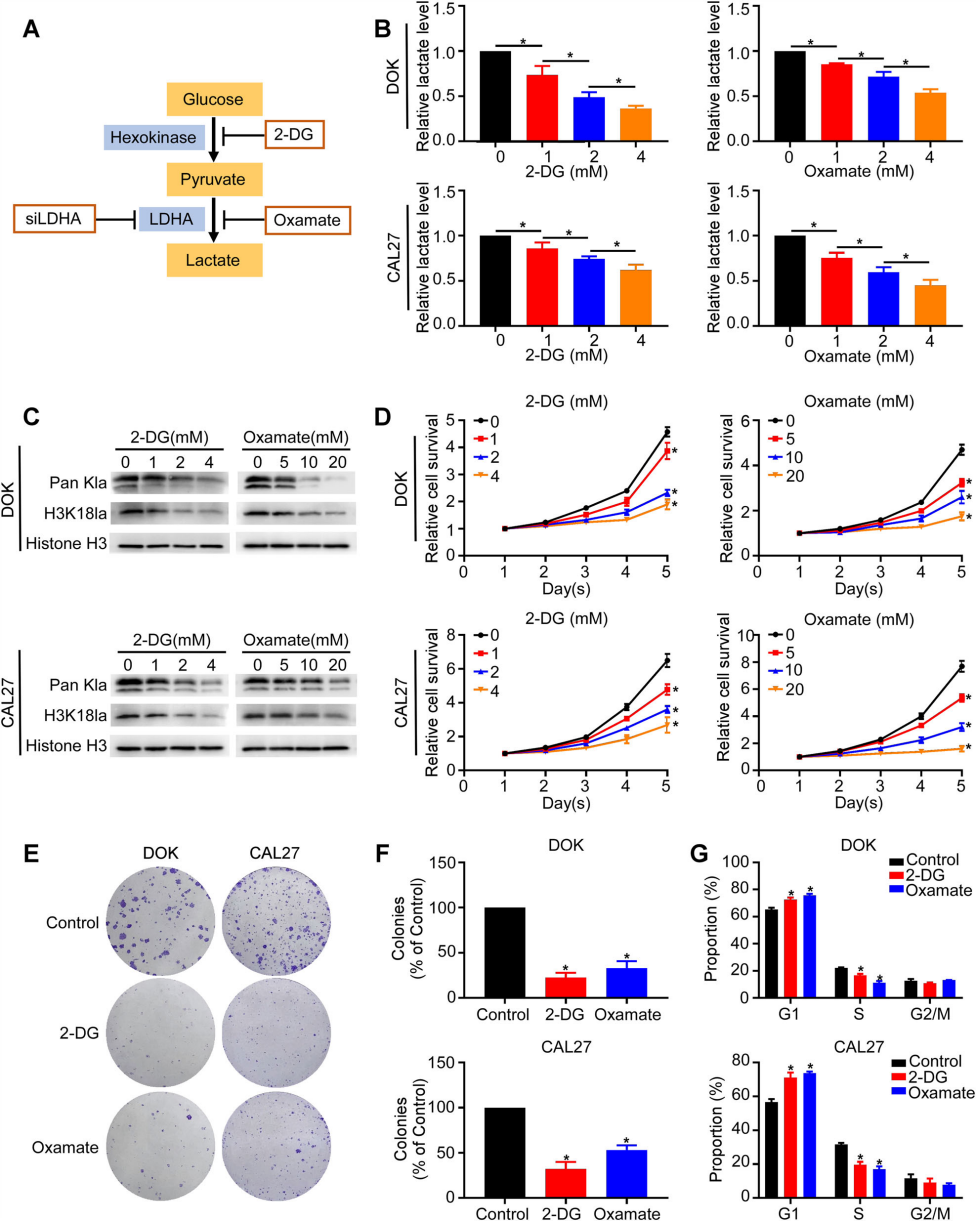
Suppressive effects of glycolysis inhibitors on the proliferation of OLK and OSCC cells. **A** Schematic diagram of strategies to inhibit histone lactylation. **B**–**G** DOK and CAL27 cells were treated with the glycolysis inhibitor 2-DG (0-4 mM) or oxamate (0-20 mM) for 24 h. Intracellular lactate levels were measured using a lactate colorimetric kit (**B**). Pan Kla and H3K18la levels were assessed using western blotting assays (**C**). Cell growth was measured using CCK-8 assay (**D**) and colony formation assay (**E**, **F**). The cell cycle distribution was determined by flow cytometry (**G**). Each experiment was performed in triplicate. Error bars, mean ± SD; ^*^P < 0.05.

To evaluate the role of histone lactylation in oral carcinogenesis, we selected DOK (OLK) [25] and CAL27 (OSCC) cells [26]. Here, lactate production and histone lactylation were attenuated by (1) glycolysis inhibitors [2-deoxy-D-glucose (2-DG) and oxamate] and (2) siRNA targeting LDHA [27] (Fig. 2A). Treatment with glycolysis inhibitors reduced intracellular lactate levels in a dose-dependent manner (Fig. 2B), accompanied by decreased Pan Kla and H3K18la levels (Fig. 2C) in DOK and CAL27 cells. We subsequently found that reduced histone lactylation significantly inhibited the viability, colony formation abilities, and migration and invasion abilities of DOK and CAL27 cells (Figs. 2D–F and S1A, B). A greater proportion of these cells were arrested in the G0/G1 phase than the cells in the control group (Figs. 2G and S1C). The proportion of apoptotic cells increased upon treatment with 2-DG and oxamate (Fig. S1D).

Since 2-DG and oxamate may have other effects independent of the suppression of lactate production and lactylation, we silenced LDHA to inhibit histone lactylation. Compared with NC group, knockdown of LDHA potently reduced the intracellular lactate content, ECAR, and Pan Kla and H3K18la levels, which were obviously increased after Nala treatment (Fig. S2A–D). In addition, LDHA knockdown inhibited the viability, colony formation abilities, and migration and invasion abilities of DOK and CAL27 cells, and Nala addition attenuated the suppressive effects of LDHA silencing (Figs. S2E, F and S3A, B). The silencing of LDHA induced G0/G1 cell cycle arrest and apoptosis, and Nala significantly decreased cell arrest at the G0/G1 phase and apoptosis (Fig. S3C, D). Importantly, we investigated the association between LDHA levels and prognosis in OSCC patients using TCGA database. Kaplan-Meier survival analysis revealed that low LDHA expression was linked to prolonged overall survival in OSCC patients (*P* = 0.049; Fig. S4). Collectively, these data suggest that histone lactylation plays a critical role in the initiation of OSCC, while inhibiting histone lactylation may exhibit potential antitumor activity.

### Inhibition of histone lactylation suppresses OSCC tumorigenesis in vivo

**Fig. 3:**
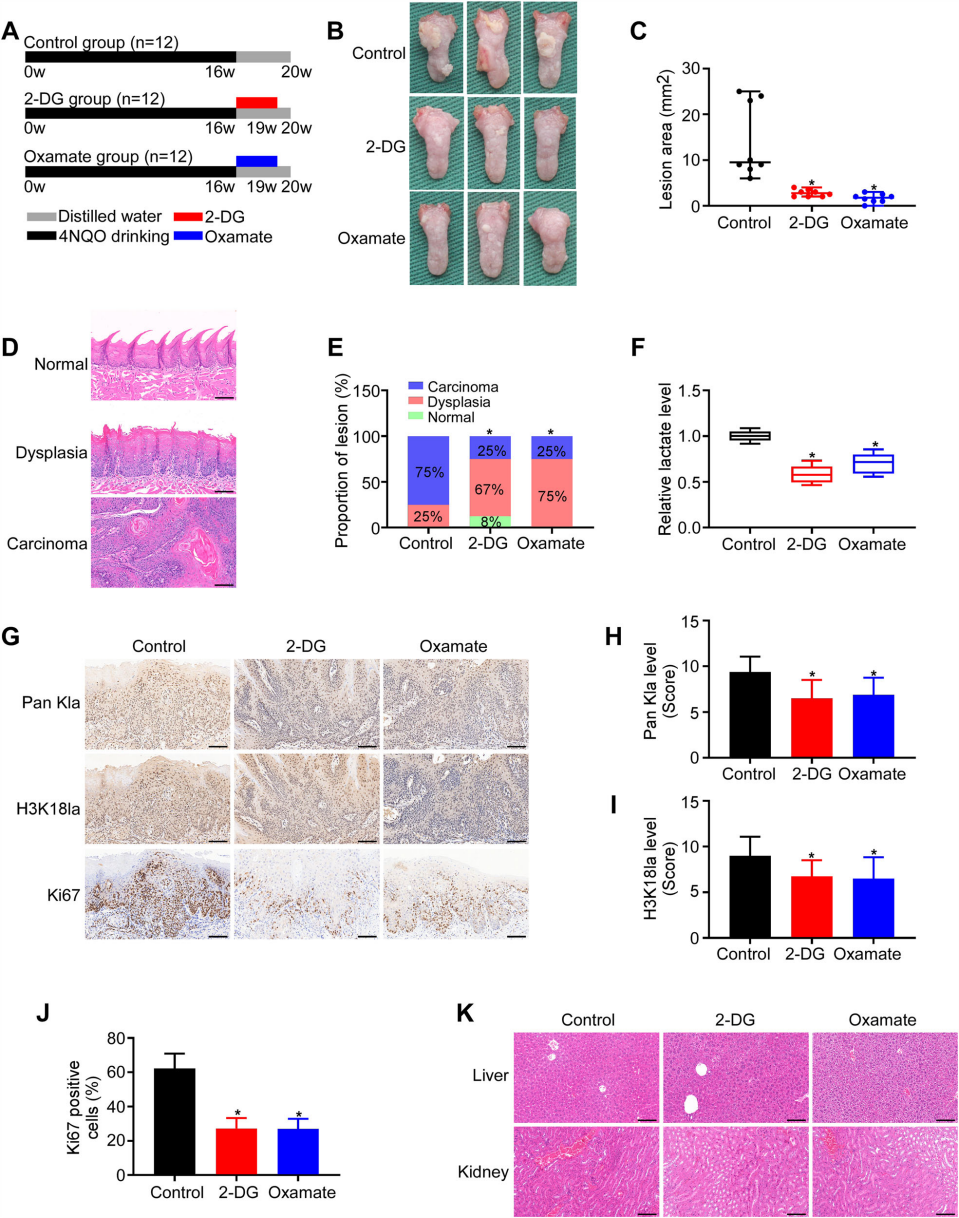
Inhibition of histone lactylation suppresses OSCC tumorigenesis in vivo. **A** Schematic diagrams displaying the experimental strategies. **B** Representative images of tongues from each group. **C** Quantification of lesion size in different groups. **D** Representative HE stained images of tissues with various pathological grades, including normal epithelium, dysplasia, and carcinoma. **E** Incidence rates of normal, dysplasia, and carcinoma lesions in the three groups. **F** Lactate content in the lesion areas. The representative images (×200) (**G**) and quantification analysis of Pan Kla (**H**), H3K18la (**I**), and Ki67 (**J**) protein levels were measured by immunohistochemical staining. Scale bar: 100 µm. **K** HE stained images of kidney and liver tissues from different groups. Scale bar: 100 µm. Error bars, mean ± SD; ^*^P < 0.05.

To further examine the biological role of histone lactylation in OSCC initiation, a 4NQO-induced mouse tongue carcinogenesis model was established [28]. We started administration of glycolysis inhibitors (2-DG and oxamate) (500 mg/kg) or vehicle control twice per week via intraperitoneal injection at week 17 (Fig. 3A). When the experiment was stopped at week 20, we found that 2-DG or oxamate significantly reduced the lesion area on the tongue surface (Fig. 3B, C). Each tongue was pathologically diagnosed and classified into normal, dysplasia, and cancer regions (Fig. 3D). Compared with that in the control group, the carcinogenesis rate in both the 2-DG- and oxamate-treated groups markedly decreased (Fig. 3E).

We assessed the effects of the suppression of glycolysis on lactate generation and lactylation in the 4NQO-induced carcinogenesis mouse model. As expected, lactate production in the lesions was decreased after glycolysis inhibitor treatment (Fig. 3F). In addition, lesions with reduced Pan Kla and H3K18la staining intensities were observed in the 2-DG- and oxamate-treated groups (Fig. 3G–I). Immunostaining for the proliferation marker Ki-67 further revealed decreased expression in both treatment groups (Fig. 3G, J), indicating suppressed epithelial proliferation. HE staining revealed that the glycolysis inhibitors exhibited no significant hepatorenal toxicity (Fig. 3K). Taken together, these findings suggest that the inhibition of glycolysis decreases the carcinogenesis rate, reduces lactate production and lactylation, and inhibits cell proliferation, suggesting an inhibitory effect on OSCC carcinogenesis in the 4NQO-induced mouse model.

### H3K18la activates TK1 transcription in OSCC tumorigenesis

**Fig. 4:**
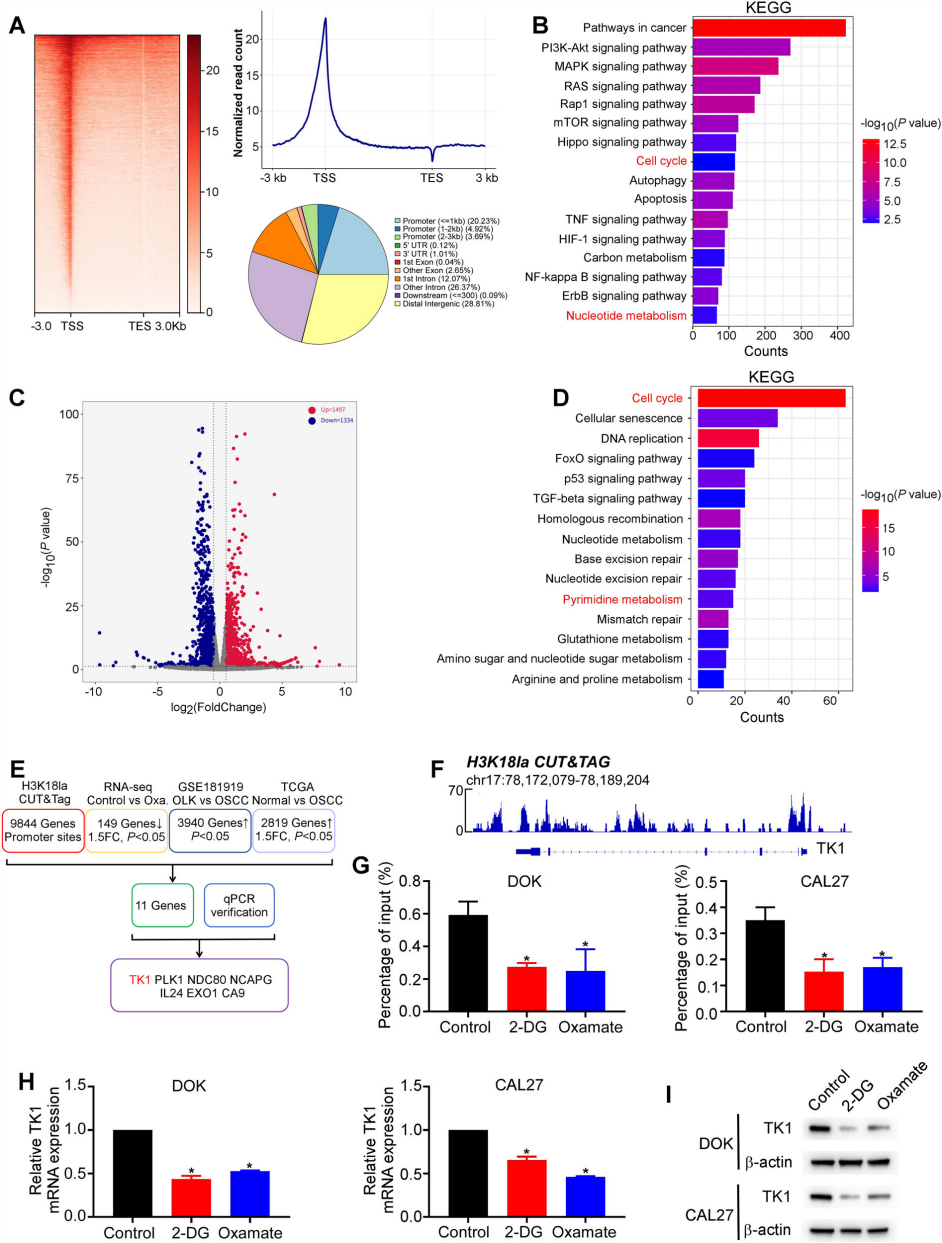
H3K18la activates the transcription of TK1. **A** CUT&Tag was performed with H3K18la antibodies in DOK cells. H3K18la could be enriched in the promoter regions of numerous genes. **B** KEGG analysis was performed in H3K18la-related genes. DOK cells were treated with oxamate (20 mM) for 24 h and gathered for RNA-seq to identify downstream genes of lactylation. Volcano plot of the DEGs in RNA-seq (**C**). KEGG analysis of the DEGs in RNA-seq (**D**). **E** CUT&Tag, RNA-seq, GSE181919, and TCGA data were combined to determine the downstream targets of H3K18la. **F** Integrative Genomics Viewer tracks of CUT&Tag showing enrichment of H3K18la in the TK1 promoter region. DOK and CAL27 cells were treated with 2-DG (4 mM) or oxamate (20 mM) for 24 h. DNA fragments were immunoprecipitated with the H3K18la antibody and analyzed by real-time RT-PCR (**G**). TK1 mRNA and protein levels were measured using real-time RT-PCR (**H**) and western blotting assays (**I**), respectively. Each experiment was performed in triplicate. Error bars, mean ± SD; ^*^P < 0.05.

Next, we attempted to determine how H3K18la influences OSCC initiation. Histone lactylation, a novel epigenetic modification, directly regulates gene transcription from chromatin [27]. Therefore, we used an anti-H3K18la antibody to perform a CUT&Tag assay. As shown in Fig. 4A, H3K18la was enriched in the promoter regions of numerous genes. Moreover, Kyoto Encyclopedia of Genes and Genomes (KEGG) analysis indicated that H3K18la-related genes identified by CUT&Tag sequencing were involved in the cell cycle, nucleotide metabolism, and pathways in cancer (Fig. 4B). We also performed RNA-seq to identify potential target genes regulated by lactylation. In DOK cells, compared with the control group, oxamate treatment upregulated 1497 genes and downregulated 1334 genes (Fig. 4C). KEGG analysis of these DEGs also revealed enrichment in the cell cycle and pyrimidine metabolism (Fig. 4D).

By overlapping the gene sets identified from CUT&Tag, RNA-seq, GSE181919, and TCGA-OSCC datasets, 10 genes were selected (Fig. 4E). Among these candidate genes, TK1 has been reported to function as an oncogene in several cancers and is associated with pyrimidine metabolism and the cell cycle [29]. Therefore, we chose to concentrate on TK1, the enzyme responsible for dTTP synthesis. To verify that H3K18la activates TK1 transcription, we first observed notable enrichment of the H3K18la signal in TK1 promoter regions (Fig. 4F). ChIP-qPCR confirmed that H3K18la was enriched in the TK1 promoter and that glycolysis inhibitors decreased this enrichment (Fig. 4G). As expected, both the mRNA and protein levels of TK1 were significantly decreased after treatment with glycolysis inhibitors (Fig. 4H, I). To explore how pyrimidine metabolism is altered by lactylation, we further examined whether the levels of dNTPs were affected using mass spectrometry. The levels of all four dNTPs, dATP, dTTP, dCTP, and dGTP, were significantly decreased by glycolysis inhibitors compared with those in the control group (Fig. S5). The decrease in the level of dTTP was the most significant, while the changes in dATP, dCTP and dGTP were relatively small. Additionally, lactate is transported across the cell membrane by monocarboxylate transporters (MCTs) [12]. Notably, the mRNA levels of MCT1 (*P* = 0.005, *R* = 0.159; Fig. S6A) and MCT3 (*P* = 0.002, *R* = 0.178; Fig. S6B) were positively correlated with TK1 expression in TCGA-OSCC cohort. Hence, these findings suggest that the transcription of TK1 is positively regulated by H3K18la.

### TK1 is a novel oncogene that contributes to OSCC carcinogenesis

**Fig. 5:**
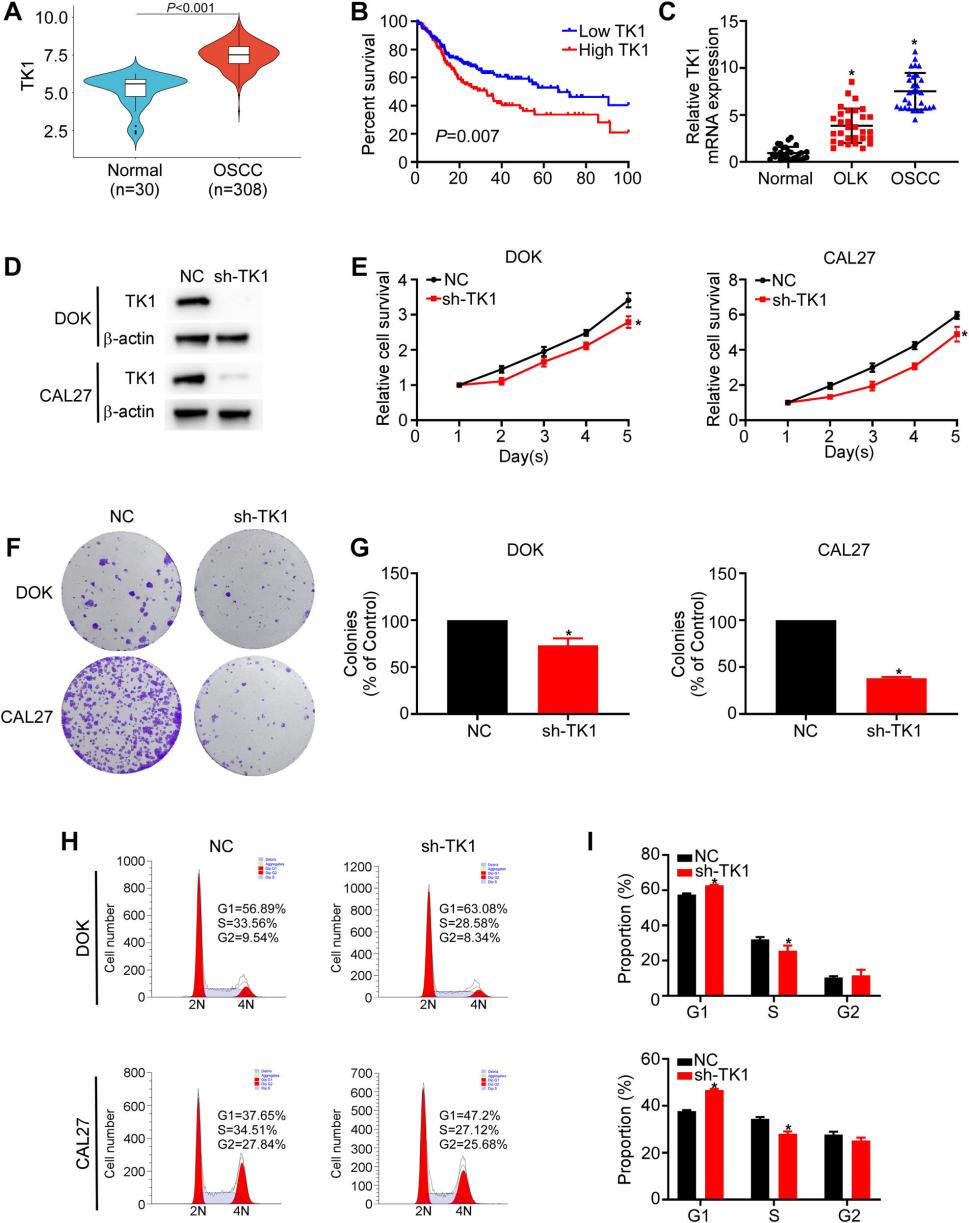
TK1 is a novel oncogene that contributes to OSCC carcinogenesis. **A** TK1 mRNA levels in normal (*n* = 30) and OSCC (*n* = 308) tissues were analyzed using the data from TCGA-OSCC cohort. **B** Kaplan–Meier survival analysis was performed to analyze overall survival based on TK1 mRNA levels (Low TK1 versus High TK1) in patients with OSCC in TCGA cohort (*n* = 308). **C** TK1 mRNA levels in normal (*n* = 30), OLK (*n* = 30), and OSCC (*n* = 30) tissues were measured using real-time RT-PCR. **D**–**I** DOK and CAL27 cells stably expressing NC or sh-TK1 were used to assess the effects of TK1 silencing on cell proliferation. The TK1 protein levels were measured using western blotting assays (**D**). Cell growth was evaluated using CCK-8 assay (**E**) and colony formation assay (**F**, **G**). The cell cycle distribution was measured using flow cytometry (**H**, **I**). Each experiment was performed in triplicate. Error bars, mean ± SD; ^*^P < 0.05.

Since H3K18la directly regulates TK1, we next investigated its function in OSCC tumorigenesis. First, we analyzed TK1 mRNA levels in an OSCC cohort in TCGA database. The results revealed that TK1 expression was significantly higher in OSCC tissues (*n* = 308) than in normal epithelial tissues (*n* = 30) (*P* < 0.001; Fig. 5A). Kaplan-Meier survival analysis revealed that OSCC patients with high TK1 mRNA levels had poorer overall survival than those with low TK1 expression did (*P* = 0.007; Fig. 5B). We further performed real-time RT-PCR and IHC assays on human normal (*n* = 30), OLK (*n* = 30), and OSCC (*n* = 30) tissue samples. The results indicated significantly higher TK1 mRNA and protein levels in OSCC samples than in OLK and normal samples (Figs. 5C and S7).

To validate the function of TK1, we silenced its expression in DOK and CAL27 cells using shRNA (sh-TK1). Western blotting assays confirmed that sh-TK1 markedly reduced TK1 protein levels in both cell lines (Fig. 5D). CCK8 and colony formation assays revealed that TK1 knockdown decreased the viability and colony formation abilities of DOK and OSCC cells (Fig. 5E–G). In addition, more cells were arrested in the G0/G1 phase in the TK1 knockdown group than in the control group (Fig. 5H, I). A xenograft tumor model was used to investigate the role of TK1 in OSCC in vivo. In contrast to tumors with the NC group, tumors with the sh-TK1 group had significantly smaller volumes, slower growth rates, and lower weights (Fig. S8). Taken together, these data indicate that TK1 functions as an oncogene and that TK1-mediated pyrimidine biosynthesis may be a promising metabolic target in OSCC initiation.

### H3K18la promotes oral carcinogenesis through TK1

**Fig. 6:**
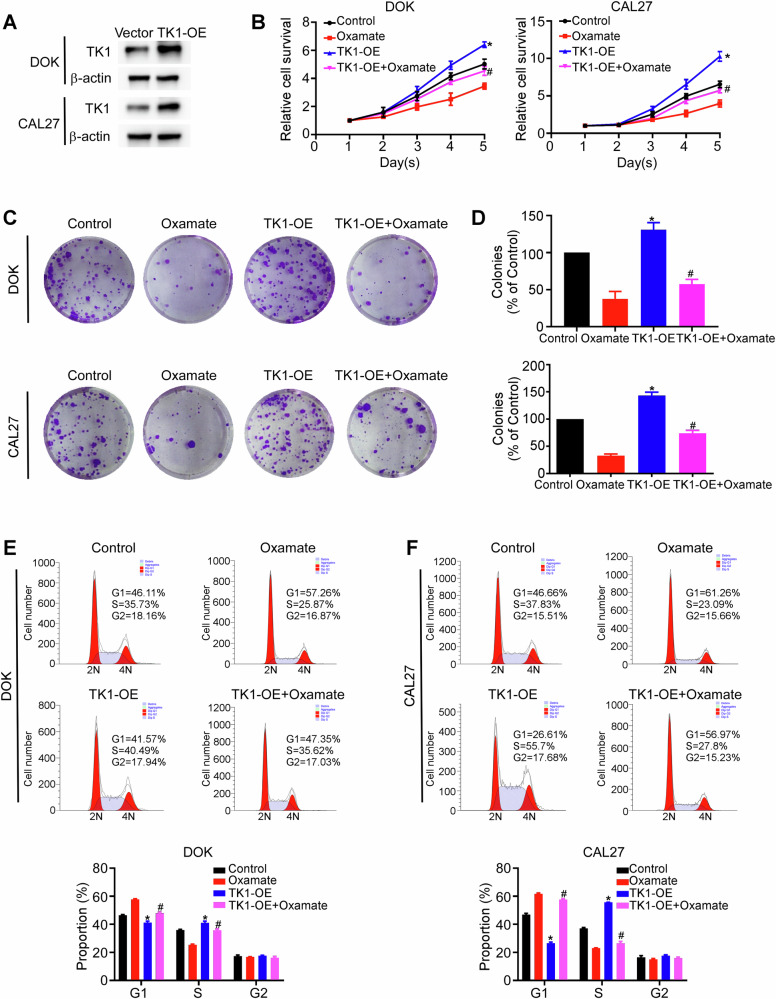
The anticancer effects of glycolysis inhibitors are partially reversed by TK1. DOK and CAL27 cells stably overexpressing vector or TK1 were treated with oxamate (20 mM). The efficiency of TK1 overexpression was determined by western blotting assays (**A**). Cell viability was measured using CCK-8 assay (**B**). Colony formation ability was determined using colony formation assay (**C**, **D**). The cell cycle distribution was measured using flow cytometry (**E**, **F**). Each experiment was performed in triplicate. Error bars, mean ± SD; ^*^P < 0.05 compared with Control; ^#^*P* < 0.05 compared with Oxamate.

After confirming that H3K18la regulates TK1 expression, we sought to determine whether TK1 overexpression could reverse the tumor suppressive effects induced by the inhibition of histone lactylation. DOK and CAL27 cells were transfected to overexpress TK1 (Fig. 6A) and treated with the glycolysis inhibitor oxamate. Compared with the negative control, TK1 overexpression increased the proliferative capacity of DOK and CAL27 cells and promoted the G1/S transition. Consistent with the findings above, oxamate suppressed the growth of DOK and CAL27 cells and induced G0/G1 arrest. Notably, TK1 overexpression in the presence of oxamate attenuated the anticancer effects of oxamate, reversing growth inhibition and G0/G1 cell cycle arrest (Fig. 6B–F). These results illustrate that H3K18la contributes to carcinogenesis, at least in part, through the regulation of TK1.

### Downregulation of TK1 inhibits the Wnt signaling pathway through RhoA

**Fig. 7:**
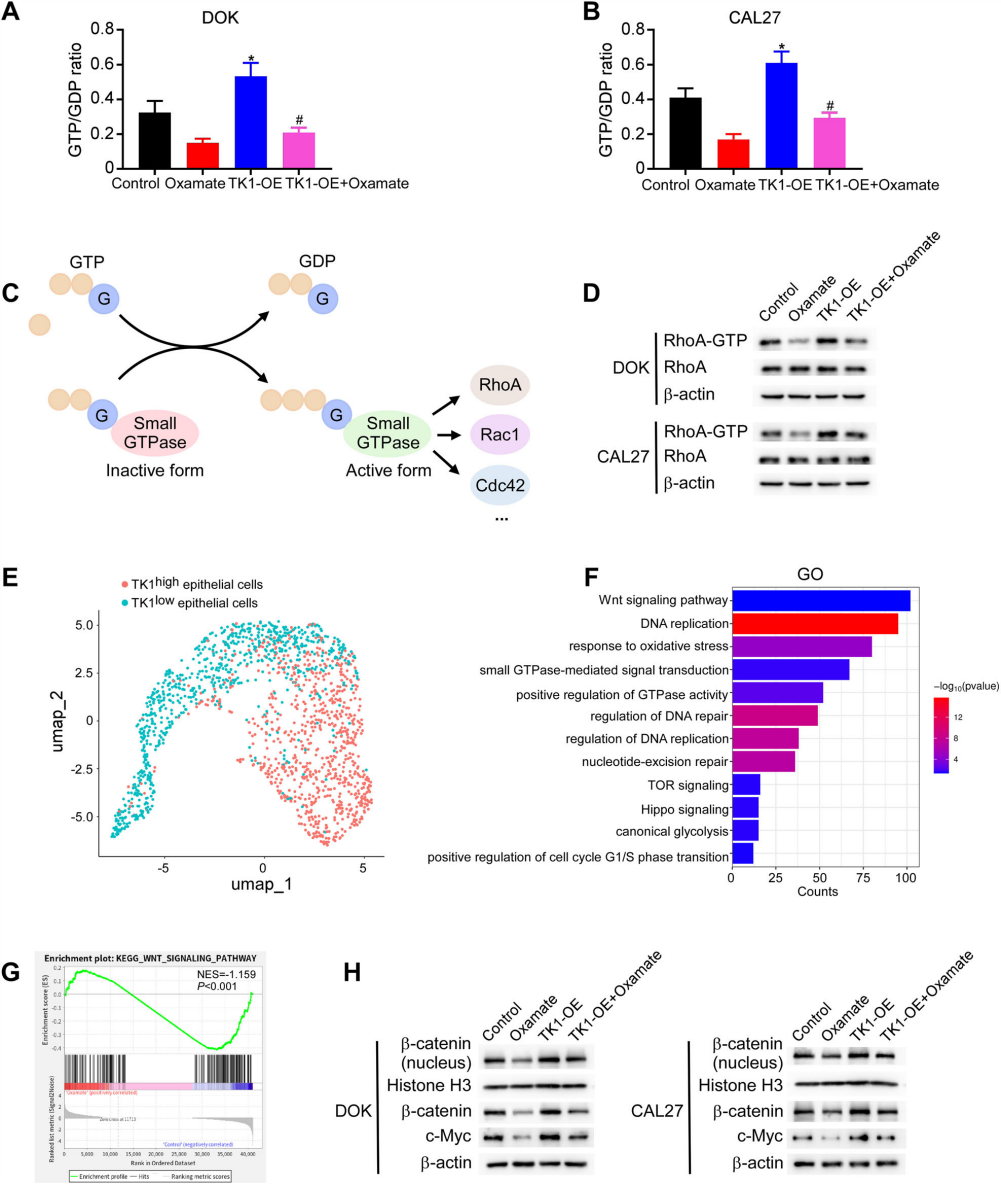
Downregulation of TK1 inhibits the Wnt signaling pathway through RhoA. **A** Diagram illustrating the GTPase cycle between the GDP-bound and GTP-bound conformations. DOK and CAL27 cells stably overexpressing vector or TK1 were treated with oxamate (20 mM). GTP/GDP ratios were subsequently measured using mass spectrometry (**B**, **C**). GTP-bound RhoA levels were detected using GTPase pull-down assays and western blotting assays. Total protein levels were assessed from whole-cell lysates (**D**). **E** UMAP plot of the clusters of TK1^high^ and TK1^low^ epithelial cells based on TK1 expression. **F** Barplot for GO analysis results of DEGs. **G** GSEA comparing the oxamate-treated group and the control group. **H** Western blotting analysis for the downstream proteins of H3K18la in oxamate-treated cells with or without TK1 overexpression. Each experiment was performed in triplicate. Error bars, mean ± SD; ^*^P < 0.05.

GTP plays a crucial role in protein synthesis and is essential for rapidly dividing tumor cells. TK1 knockdown could lead to a reduction in the GTP/GDP ratio because of the decreased activation of ribonucleotide reductase [30]. Therefore, we investigated the GTP/GDP ratios in cells treated with glycolysis inhibitors using mass spectrometry. The results indicated that the GTP/GDP ratios were obviously decreased in both DOK and CAL27 cells following treatment with 2-DG or oxamate (Fig. S9), which could be rescued by TK1 overexpression (Fig. 7A, B). Changes in the GTP/GDP ratios have been shown to be critical for GTPase activity [31, 32]. The activities of Rho GTPases, particularly those of the family (including most studied members RhoA, Rac1, and Cdc42), are closely associated with tumor growth in various cancers, including OSCC (Fig. 7C) [33]. TK1 knockdown has been shown to reduce cancer progression by deregulating RhoA activity through alterations in the GTP/GDP ratio [30]. Thus, we investigated whether H3K18la could regulate RhoA activity via TK1. As expected, oxamate-treated cells displayed reduced levels of GTP-bound RhoA, whereas TK1 overexpression rescued RhoA activity (Fig. 7D).

Given the above results, we sought to identify the signaling pathway affected by the H3K18la-TK1 axis in the regulation of RhoA activity. The epithelial cells during OSCC initiation could be divided into two populations characterized by TK1^high^ and TK1^low^ (Fig. 7E). We then identified DEGs between the TK1^high^ and TK1^low^ groups. We then identified DEGs between the TK1^high^ and TK1^low^ groups. GO analysis was performed to detect signaling pathways involved in OSCC tumorigenesis. The results revealed that there was enrichment of the DEGs in DNA replication, G1/S phase transition, glycolysis, and pathways related to carcinogenesis, such as Wnt and Hippo signaling (Fig. 7F). Among these pathways, RhoA is known to activate Wnt/β-catenin signaling by promoting β-catenin nuclear translocation [34]. In addition, GSEA of DOK cells treated with and without oxamate revealed that lactylation may inhibit Wnt signaling (Fig. 7G). Activation of the canonical Wnt pathway induces glycolysis through the nuclear translocation of β-catenin, where it interacts with T-cell factor/lymphoid enhancer factor to trigger the transcription of many target genes, including c-Myc [35]. To confirm that H3K18la regulates the Wnt/β-catenin pathway through TK1, we examined its effect on nuclear β-catenin in oxamate-treated and TK1-overexpressing cells. Western blotting assays revealed that the oxamate-induced decrease in nuclear β-catenin and c-Myc expression was reversed by TK1 overexpression in both DOK and CAL27 cells (Fig. 7H). These results indicate that the H3K18la-TK1 axis modulates the Wnt pathway through the regulation of RhoA activity.

### Glycolysis/H3K18la/TK1/β-catenin forms a positive feedback loop

**Fig. 8:**
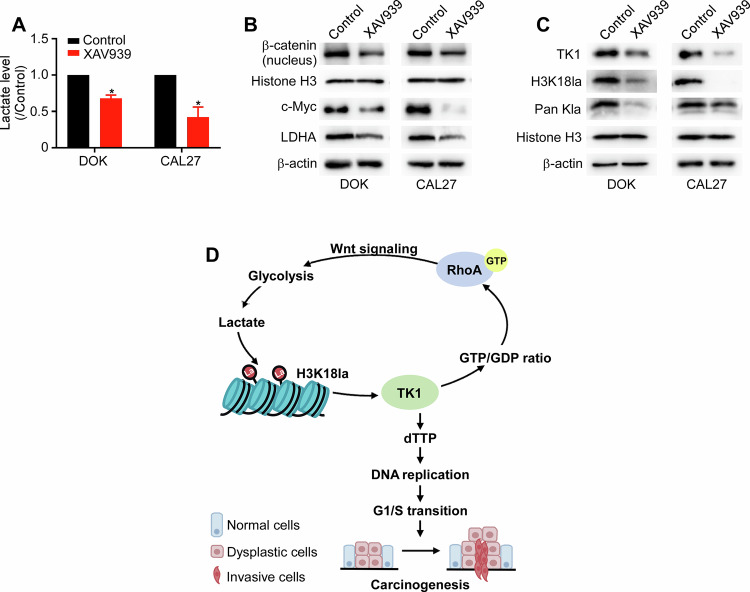
Glycolysis/H3K18la/TK1/β-catenin forms a positive feedback loop. DOK and CAL27 cells were treated with XAV939 (10 μM) for 24 h. Intracellular lactate levels were measured by a lactate colorimetric kit (**A**). Western blotting analysis of nuclear β-catenin, c-Myc, and LDHA protein expression (**B**). Western blotting assays were used to assess Pan Kla, H3K18la, and TK1 protein levels (**C**). **D** Schematic summary of the glycolysis/H3K18la/TK1/β-catenin feedback loop. Each experiment was performed in triplicate. Error bars, mean ± SD; ^*^P < 0.05.

**Fig. 9:**
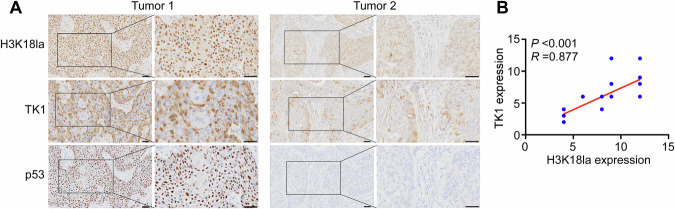
Clinicopathological relevance of H3K18la, TK1, and p53. **A** The representative images (×200 and ×400) of H3K18la, TK1 and p53 protein levels in OSCC (*n* = 30) tissues were assessed by immunohistochemical staining. **B** Correlation between H3K18la and TK1 protein levels in our cohort was determined using Spearman correlation analysis. Scale bar: 50 µm.

Previous studies have confirmed an association between the Wnt/β-catenin signaling pathway and aerobic glycolysis in cancer cells [36]. Oncogenes such as c-Myc, a Wnt/β-catenin target gene, are considered “master regulators” of this process because they directly control various genes involved in glucose metabolism, including LDHA [35]. To investigate whether Wnt/β-catenin also regulates glycolysis and influences H3K18la to establish a positive feedback loop, DOK and CAL27 cells were treated with the β-catenin-specific inhibitor XAV939 in vitro. XAV939 treatment significantly reduced the intracellular lactate levels (Fig. 8A). Western blotting assays revealed a marked decrease in the levels of nuclear β-catenin, c-Myc, and the key glycolytic enzyme LDHA (Fig. 8B). Notably, the levels of Pan Kla, H3K18la, and TK1 also decreased significantly (Fig. 8C). Overall, inhibition of Wnt signaling in DOK and CAL27 cells reduces glycolysis levels and disrupts the glycolysis/H3K18la/TK1/β-catenin positive feedback loop (Fig. 8D). Immunohistochemical assays were conducted to examine the levels of H3K18la and TK1 in our own clinical cohort. Spearman correlation analysis revealed that the H3K18la expression was positively correlated with TK1 expression in OSCC samples (Fig. 9). Given the relevance of p53 in OSCC and in the context of lactate metabolism and histone lactylation, we conducted IHC for p53 in OSCC specimens in our cohort. Furthermore, we divided these 30 patients into high (*n* = 15) and low (*n* = 15) H3K18la expression groups based on the median value of the SI (SI = 8.5). The results showed that p53 status was associated with the H3K18la protein expression (Fig. 9 and Table 1).

**Table 1:** Relationship between p53 status and H3K18la protein levels in patients with OSCC.

Variables	H3K18la expression			
	High (%)	Low (%)	Total	*P* value^a^
p53 status (*n* = 30)				
Positive	14 (87.5)	2 (12.5)	16	<0.001^*^
Negative	1 (7.1)	13 (92.9)	14	

^a^ Fisher’s exact test. ^*^, *P* < 0.05.

**Fig. 10:**
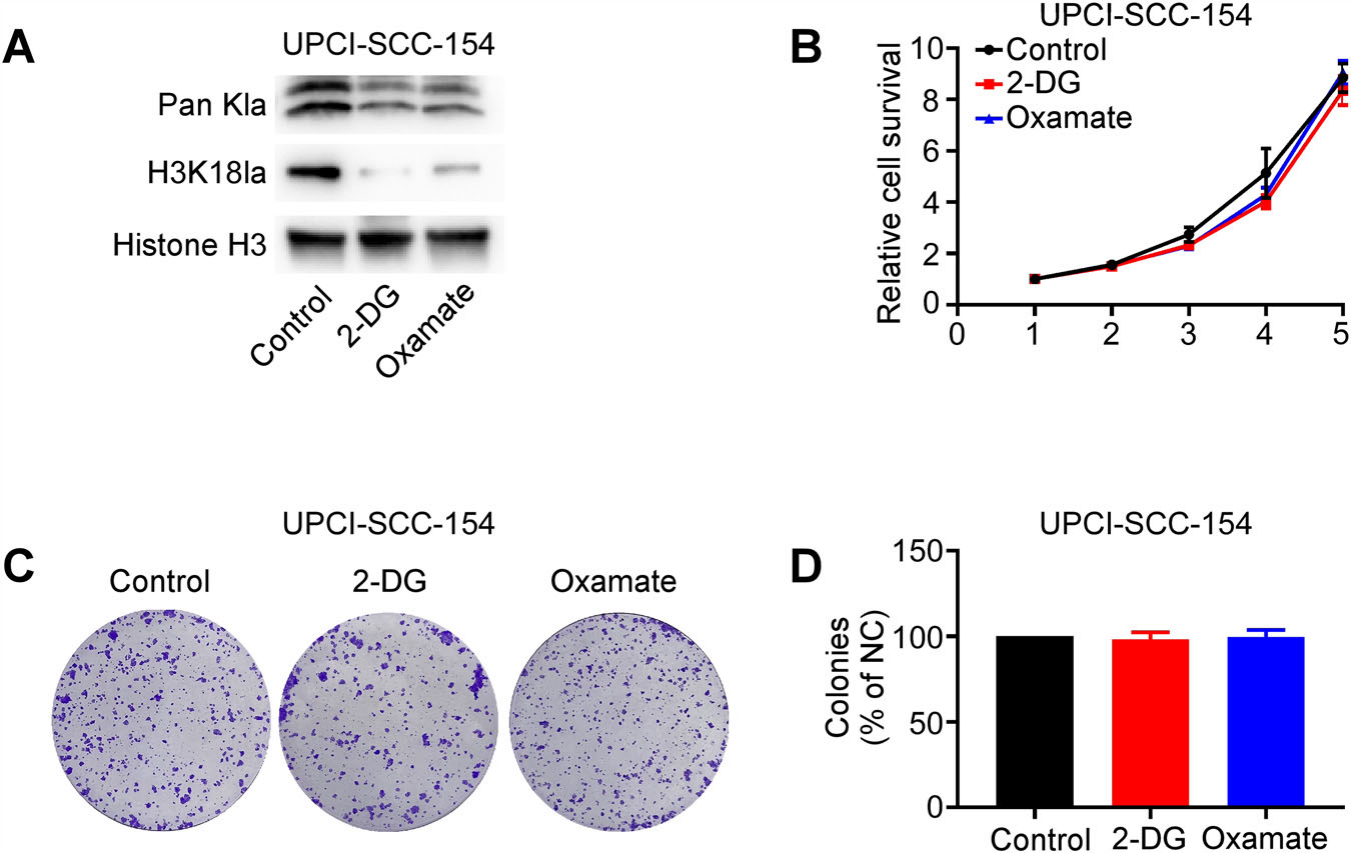
Effects of glycolysis inhibitors on the proliferation of HPV-positive OSCC cells. UPCI-SCC-154 cells were treated with the glycolysis inhibitor 2-DG (4 mM) or oxamate (20 mM) for 24 h. Pan Kla and H3K18la levels were assessed using western blotting assays (**A**). Cell growth was measured using CCK-8 assay (**B**) and colony formation assay (**C**, **D**). Each experiment was performed in triplicate. Error bars, mean ± SD; ^*^P < 0.05.

Given the established correlation between HPV infection and OSCC, we conducted parallel experiments in the HPV-positive OSCC cell line UPCI-SCC-154 [16, 37, 38]. Treatment with glycolysis inhibitors reduced Pan Kla and H3K18la levels (Fig. 10A) in UPCI-SCC-154 cells, but reduced histone lactylation did not significantly inhibit the viability and colony formation abilities of UPCI-SCC-154 cells (Fig. 10B–D). This indicates that the role of H3K18la in HPV-negative and HPV-positive OSCC cells is different [16, 38].

## Discussion

Our investigation revealed a significant increase in lactate accumulation within the microenvironments of OLK and OSCC, which subsequently served as a substrate for histone lactylation, particularly H3K18la, which emerged as a key driver of OSCC tumorigenesis. Moreover, H3K18la activated TK1 transcription, thereby promoting pyrimidine biosynthesis, G1/S transition, and tumorigenesis in OSCC. Intriguingly, inhibition of the Wnt pathway resulted in reduced LDHA expression, leading to decreased lactate production and histone lactylation levels and the formation of a feedback loop. In summary, our study provides novel insights into the role of histone lactylation in OSCC pathogenesis.

OSCC is characterized by uncontrolled cell growth. Cancer cells can adapt to a changing microenvironment by rewiring metabolic processes to generate energy and activate oncogenic signaling pathways [17]. Glycolysis plays a critical role in the metabolic reprogramming of OSCC cells and promotes cell survival, proliferation, and malignant progression [39]. Lactate was previously regarded as a byproduct of glycolytic metabolism. In recent years, research has shown that lactate-derived histone lactylation is a newly defined PTM that closely links the Warburg effect to epigenetic modification [8]. Increased histone lactylation has been shown to influence tumor cell biology in several cancers, such as esophageal squamous cell carcinoma [40]. Nevertheless, studies on the role of histone lactylation in OSCC initiation have not yet been reported. In the present study, we investigated the accumulation of lactate, which acts as a substrate for histone lactylation, in the microenvironments of OLK and OSCC using scRNA-seq analysis and validation experiments. Here, we report for the first time that histone lactylation, particularly H3K18la, promotes OSCC tumorigenesis both in vitro and in vivo.

Increased histone lactylation in promoter regions promotes the expression of target genes, such as pore membrane protein 121 [11] and ubiquitin E3 ligase NEDD4 [41], in various cell types. In this study, we found that H3K18la activated TK1 transcription to promote pyrimidine metabolism and drive the G1/S transition, thereby accelerating OSCC tumorigenesis for the first time. Nucleotide synthesis and utilization represent vital metabolic dependencies in many cancer types [42, 43]. Recent studies have linked genes involved in pyrimidine metabolism to cancer progression, suggesting that targeting important enzymes and pathways involved in pyrimidine metabolism may yield anticancer effects by disrupting nucleotide synthesis and inhibiting cellular proliferation [44, 45]. TK1 is a pyrimidine salvage enzyme involved in DNA synthesis, replication, and repair. TK1 activity is absent or low in resting cells, increases during the G1/S transition, peaks in S phase, and decreases during mitosis [46, 47]. In this study, we confirmed that TK1 was highly expressed in OLK and OSCC tissues and functioned as a prognostic factor for OSCC. Using glycolysis inhibitors alongside TK1 knockdown and overexpression, we validated the role of TK1 in H3K18la-mediated oral carcinogenesis. Previous studies have reported that histone lactylation preferentially affects enzymes involved in essential metabolic pathways such as nucleotide metabolism [10], but we first identified the specific mechanism through which histone lactylation regulates pyrimidine metabolism. Further research into the epigenetic regulation of nucleotide metabolism is necessary to determine whether other histone lactylation modifications influence the expression of nucleotide metabolism enzymes.

Recently, H3K18 was identified as a crucial site of PTMs, including lactylation and acetylation. The maintenance of regular cellular metabolism and pathological activities can both cause or result in changes in the levels of lactylation and acetylation at the H3K18 region [48–50]. These alterations are frequently associated with variations in acylation substrates, metabolic substrates, and the expression or activity of lactylation-related enzymes, histone acetyltransferases and histone deacetylases [8, 51]. Di et al. reported that an increase in H3K18la may accompany a decrease in H3K18ac level during infection. This could have either antagonistic or synergistic effects on the levels of downstream target genes [52]. We suggest that potential crosstalk between H3K18la and H3K18ac in OSCC tumorigenesis could affect gene expression at the same locus. However, this topic requires further study.

Studies have shown that dTTP, the product of TK1, functions as an allosteric activator of ribonucleotide reductase, which preferentially converts GDP into dGDP. This evolutionarily conserved mechanism maintains the physiological balance of de novo-synthesized dNTP pools. dTTP-induced dGDP synthesis leads to GDP depletion, thereby altering the GTP/GDP ratio [30, 53, 54]. Our findings demonstrated that the intracellular GTP/GDP ratios were lower in cells treated with glycolysis inhibitors than in control cells, but that this effect was reversed by TK1 overexpression. An elevated GTP/GDP ratio increases the proportion of active G-coupled proteins, including RhoA [55]. Here, we showed that RhoA activity was suppressed in cells treated with oxamate but reversed by TK1 overexpression.

In this study, TCGA data analysis and RNA-seq results indicated that TK1 expression was associated with the Wnt signaling pathway. Previous research has shown that RhoA may activate Wnt signaling. In tongue squamous cell carcinoma, increased RhoA expression has been shown to activate the Wnt/β-catenin signaling pathway [56]. In addition, activated RhoA has been demonstrated to promote β-catenin accumulation in HEK293 cells in response to Wnt3A [57]. Similarly, in benign prostatic hyperplasia, RhoA activates Wnt/β-catenin signaling by enhancing β-catenin nuclear translocation and upregulating its expression through the suppression of proteasomal degradation [34]. Thus, we proposed that H3K18la could mediate the activation of Wnt signaling through TK1, which was confirmed by our results. Furthermore, activation of the Wnt/β-catenin pathway promoted the expression of c-Myc and LDHA, which increased lactate production and TK1 lactylation, thereby establishing a closed-loop feedback cycle. These findings provide insight into a novel metabolic mechanism of protein lactylation in OSCC initiation.

P53 mutations are frequent in OSCC and represent some of the earliest genomic alterations in oral carcinogenesis [58]. Mechanistically, p53 acts as an effective inhibitor of glycolysis by activating TP53-induced glycolysis and apoptosis regulator and inhibiting GLUT1 to limit lactate production [59]. Therefore, the loss of p53 function leads to a significant increase in lactate production, providing sufficient substrates for histone lactylation. Moreover, p53 regulates cell cycle checkpoints and DNA replication stress responses, which have been directly linked to TK1 activity and nucleotide metabolism via reductase regulatory subunit M2 and dNTP pool balance [60, 61]. Therefore, the interplay between p53 dysfunction and TK1 overexpression could further exacerbate replication stress and genomic instability in early OSCC lesions. When mutated, resulting in a defect in the degradation pathway, the p53 protein remains more stable and can be detected by immunohistochemistry. P53 immunohistochemistry has been widely used as a surrogate marker for p53 mutation. However, the correlation is not perfect [18, 62]. The results showed that p53 status was associated with the H3K18la protein expression, which was consistent with our hypothesis. Clarifying the precise interaction between p53 mutation and the glycolysis/H3K18la/TK1/β-catenin positive feedback loop with DNA sequencing will be an important direction for our future research [63].

Our study primarily delineates the crucial role of H3K18la in cell cycle progression. However, given the influence of p53 on cell death [64] and the observed apoptotic phenotype upon histone lactylation inhibition, it is pertinent to consider how H3K18la might intersect with apoptosis regulation. Although apoptosis-related pathways were not among the top significantly altered pathways in our RNA-seq analysis, our CUT&Tag data provide crucial mechanistic insight. They reveal that H3K18la activates the transcription of key apoptosis-related genes. In our future research, we will conduct more in-depth studies on the mechanism by which H3K18la regulates apoptosis.

It is now well-established that HPV-positive and HPV-negative OSCCs are distinct disease entities with different molecular profiles, clinical behaviors, and patient outcomes [1, 65]. One novel finding of this study is that H3K18la shows a stronger functional necessity in HPV-negative OSCC cells. This may reveal the etiological heterogeneity of OSCC. HPV-negative OSCC driven by traditional risk factors, such as tobacco and alcohol use, may be more dependent on the ‘metabolic-epigenetic’ carcinogenic pattern. For HPV-positive OSCC, its potent viral oncoproteins may have already provided sufficiently strong proliferation and survival signals, thereby relatively reducing its dependence on this metabolic-epigenetic node [66]. This requires future validation in clinical samples with clearly defined HPV status and more models, pointing the way for subsequent research [1, 65, 66].

Our study has several limitations. Firstly, further research is needed to determine whether lactylation and the metabolic effects of lactate coexist, as the biological impact of lactate metabolism cannot be completely ruled out. Secondly, the precise mechanism by which H3K18la regulates TK1 expression and its promoter binding site requires additional investigation. Thirdly, a deeper understanding of the role of histone lactylation in oral carcinogenesis may be achieved by identifying compounds that directly target lactylation sites. Finally, the link between intracellular lactate derived from glycolysis and histone lactylation is not directly demonstrated. The isotope tracing experiment needs to be carried out in future research.

In summary, we investigated histone lactylation in OLK and OSCC and found that lactate production promoted histone lactylation, particularly H3K18la, during oral carcinogenesis. H3K18la enhanced pyrimidine metabolism to promote cell proliferation by regulating TK1 expression. Furthermore, TK1 activated the RhoA/Wnt/β-catenin signaling pathway, and glycolysis was accelerated to increase the production of lactate, which further increased histone lactylation. Histone lactylation may represent a promising direction for future research and a potential therapeutic target for OSCC tumorigenesis.

## Supplementary information

Supplementary information

Original Western blots

## Data Availability

All data relevant to this research, whether generated or analyzed, are provided in this manuscript and its supplementary information. For any further inquiries, interested parties can contact the corresponding authors.
