# Supplementary material for: Histone lactylation-driven feedback loop modulates pyrimidine metabolism to promote oral carcinogenesis
Source: Cell Death Dis. 2026 Mar 19;17(1):316. doi: 10.1038/s41419-026-08580-w (PMC13039119; doi:10.1038/s41419-026-08580-w)
Supplement: Supplementary file 1 — Supplementary information [file 41419_2026_8580_MOESM1_ESM.pdf]

Title Page

Research Article

Title:

Histone lactylation-driven feedback loop modulates pyrimidine metabolism to promote oral carcinogenesis

Running title:

Promotion role of histone lactylation in OSCC initiation

Authors and Affiliations:

Yanting Wang[1,2,3*], Yanlin Geng[1,2,3], Yannan Chen[4], Haowen Zhang[4], Jingyu Liu[4], Yulin Song[4], Gang Wu[5], Tim Forouzanfar[5], Yuan Fan[1,2,3*]

[1] Department of Oral Mucosal Diseases, The Affiliated Stomatological Hospital of Nanjing Medical University, Nanjing, China.

[2] State Key Laboratory Cultivation Base of Research, Prevention and Treatment for Oral Diseases, Nanjing, China.

[3] Jiangsu Province Engineering Research Center of Stomatological Translational Medicine, Nanjing, China.

[4] Stomatological College of Nanjing Medical University, Nanjing, China.

[5] Department of Oral and Maxillofacial Surgery, Leiden University Medical Center (LUMC), Leiden, the Netherlands.

[*]Corresponding author:

Yanting Wang, The Affiliated Stomatological Hospital of Nanjing Medical University, State Key Laboratory Cultivation Base of Research, Prevention and

Treatment for Oral Diseases, Jiangsu Province Engineering Research Center of Stomatological Translational Medicine, Nanjing 210000, China; **Telephone:** +86 25 69593053; **Fax:** 025-86516414; **E-mail:** wyt@njmu.edu.cn

Yuan Fan, The Affiliated Stomatological Hospital of Nanjing Medical University, State Key Laboratory Cultivation Base of Research, Prevention and Treatment for Oral Diseases, Jiangsu Province Engineering Research Center of Stomatological Translational Medicine, Nanjing 210000, China; **Telephone:** +86 25 69593053; **Fax:** 025-86516414; **E-mail:** fanyuan@njmu.edu.cn

Figure S1

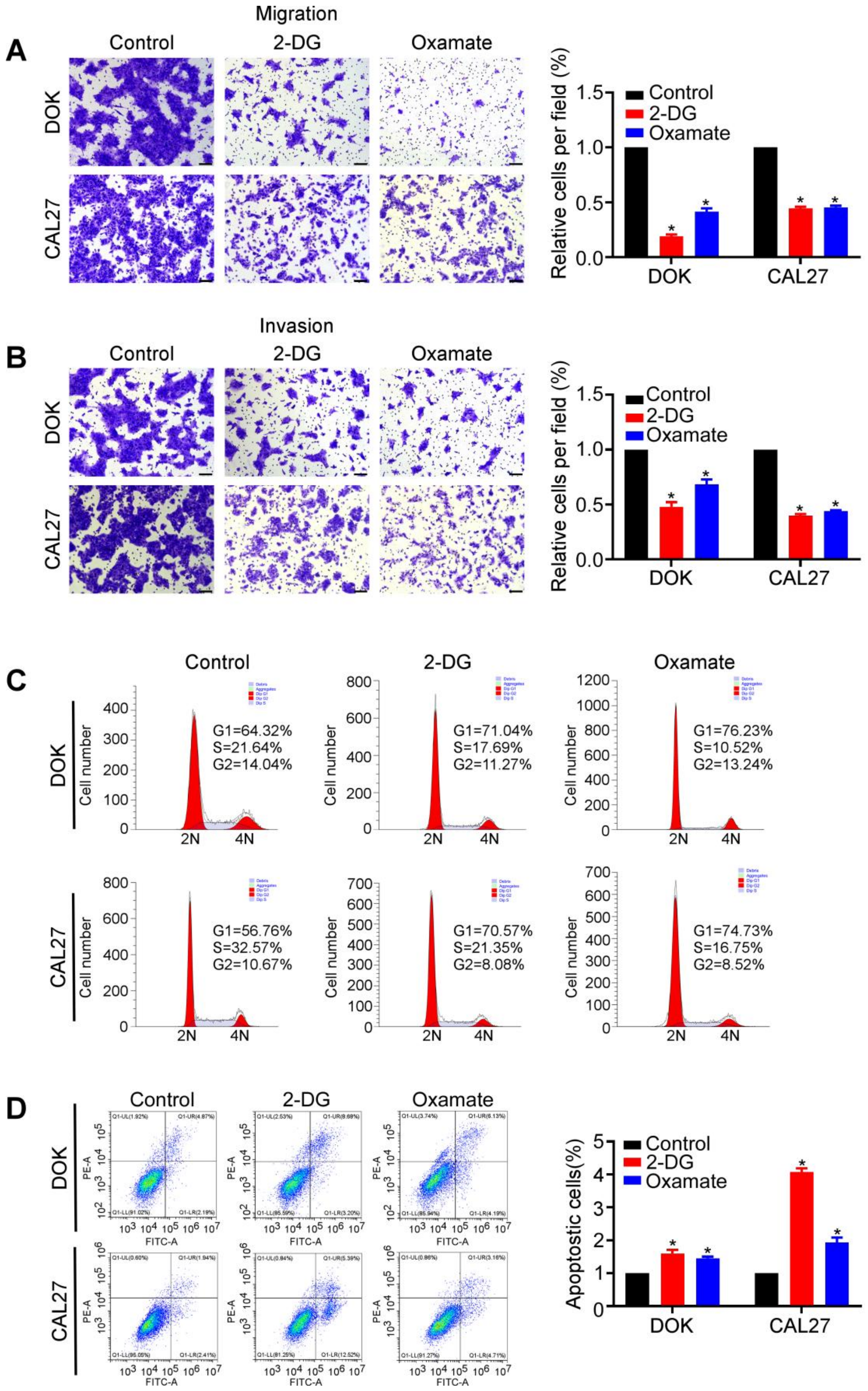

Fig. S1 Effects of glycolysis inhibitors on the migration and invasion abilities, cell cycle, and apoptosis of OLK and OSCC cells. (A-D) DOK and CAL27 cells were

treated with the glycolysis inhibitor 2-DG (4 mM) or oxamate (20 mM) for 24 h. (A-B) Migratory and invasive abilities were detected with Transwell assays without (A) and with Matrigel (B), respectively (×100). Scale bar: 100 μm. Flow cytometry of cell cycle analysis (C). Apoptosis was subsequently examined using flow cytometry (D). Each experiment was performed in triplicate. Error bars, mean ± SD; $^{*}P < 0.05$.

Figure S2

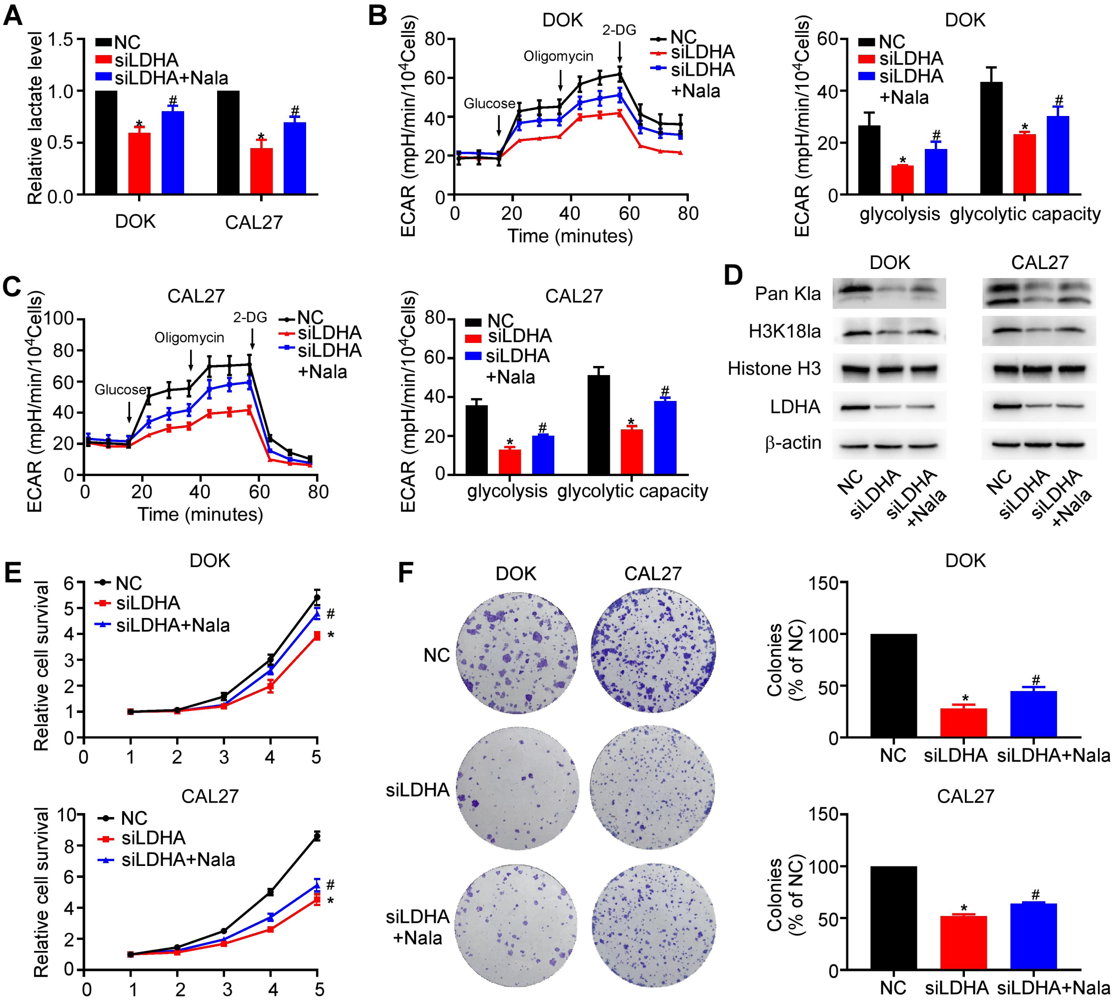

Fig. S2 Silencing LDHA reduces histone lactylation and inhibits the proliferation of OLK and OSCC cells. (A-F) DOK and CAL27 cells were transfected with LDHA siRNA (siLDHA) or negative control (NC) with or without sodium lactate (NaLa, 10 mM) treatment for 48 h. Intracellular lactate levels were measured using a lactate colorimetric kit (A). The real-time measurement of ECAR was performed by the XF96 Pro analyzer (B-C). Pan Kla and H3K18la levels were determined by western blotting assays (D). Cell growth was measured using CCK-8 assay (E) and colony formation assay (F). Each experiment was performed in triplicate. Error bars, mean ± SD; $^{*}P < 0.05$ compared with NC; $^{\#}$compared with siLDHA.

Figure S3

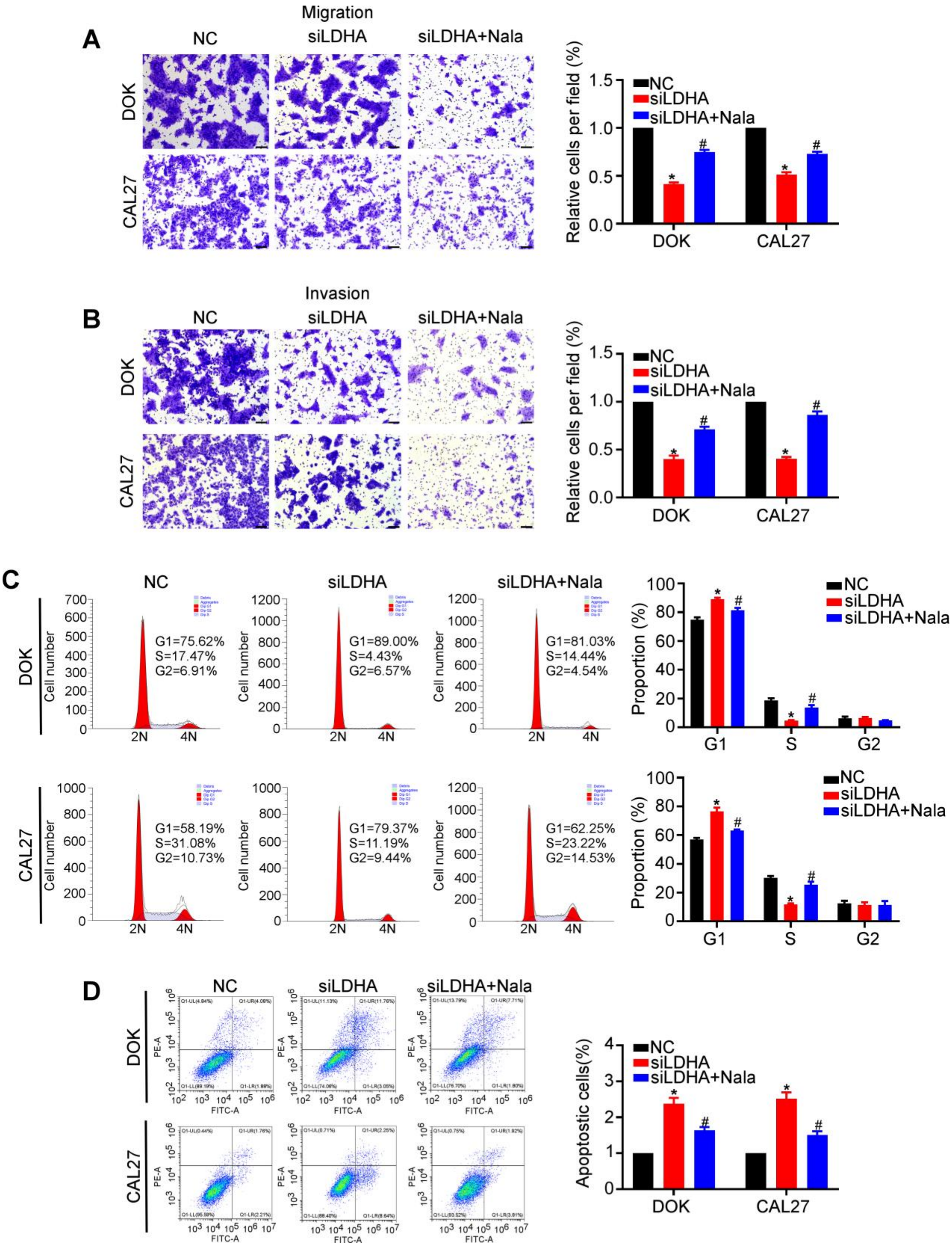

Fig. S3 Effects of silencing LDHA on the migration and invasion abilities, cell cycle, and apoptosis of OLK and OSCC cells. (A-D) DOK and CAL27 cells were transfected with LDHA siRNA (siLDHA) or negative control (NC) with or without sodium lactate (NaLa, 10 mM) treatment for 48 h. (A-B) Migratory and invasive

abilities were detected with Transwell assays without (A) and with Matrigel (B), respectively (×100). Scale bar: 100 μm. The cell cycle distribution was determined by flow cytometry (C). Apoptosis was examined using flow cytometry (D). Each experiment was performed in triplicate. Error bars, mean ± SD; $^{*}P < 0.05$.

Figure S4

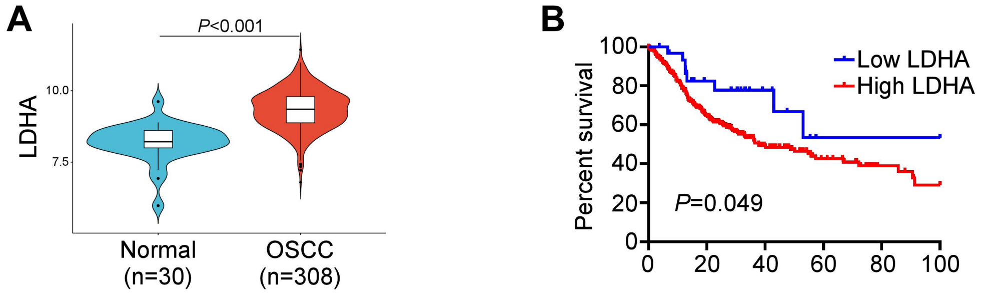

Fig. S4 LDHA upregulation is correlated with poor overall survival in OSCC. (A) LDHA mRNA levels in normal (n = 30) and OSCC (n = 308) tissues were verified using TCGA cohort. (B) Kaplan-Meier method was utilized to determine overall survival on the basis of LDHA mRNA levels (Low LDHA versus High LDHA) in patients with OSCC in TCGA cohort (n = 308).

Figure S5

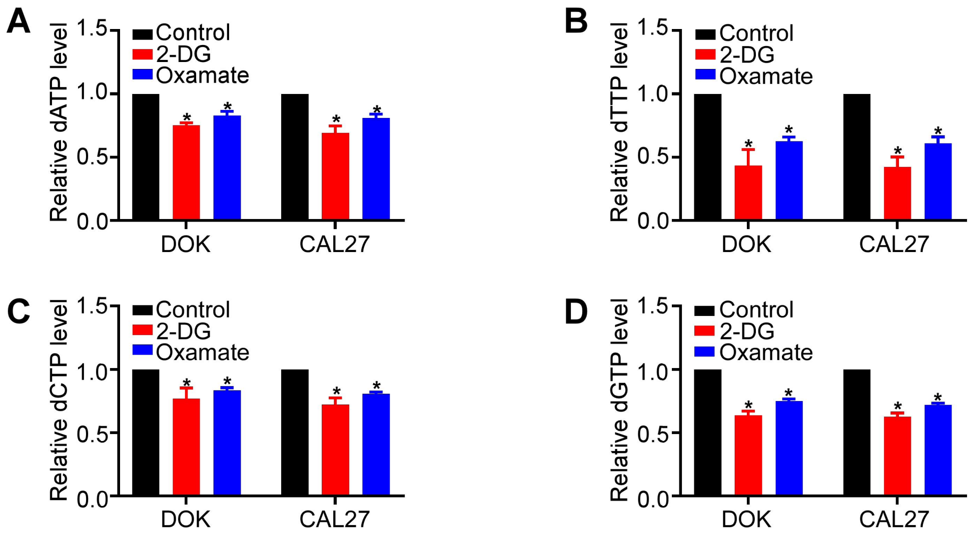

Fig. S5 Suppressive effect of glycolysis inhibitors on dNTP levels. (A-D) DOK and CAL27 cells were treated with the glycolysis inhibitor 2-DG (4 mM) or oxamate (20 mM) for 24 h. dATP (A), dTTP (B), dCTP (C), and dGTP (D) levels were detected by mass spectrometry. Each experiment was performed in triplicate. Error bars, mean ± SD; $^{*}P < 0.05$.

Figure S6

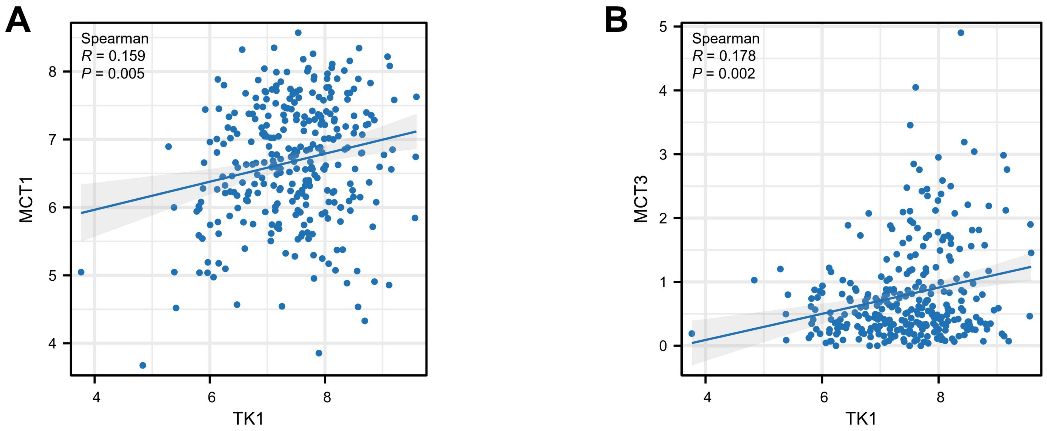

Fig. S6 MCT1 and MCT3 mRNA levels show a positive correlation with that of TK1. (A) Correlation(A) The correlation between LDHA and MCT1 mRNA levels in TCGA-OSCC cohort (n = 308) was evaluated using Spearman correlation analysis. (B) The correlation between LDHA and MCT3 mRNA levels in TCGA-OSCC cohort (n = 308) was evaluated by Spearman correlation analysis.

Figure S7

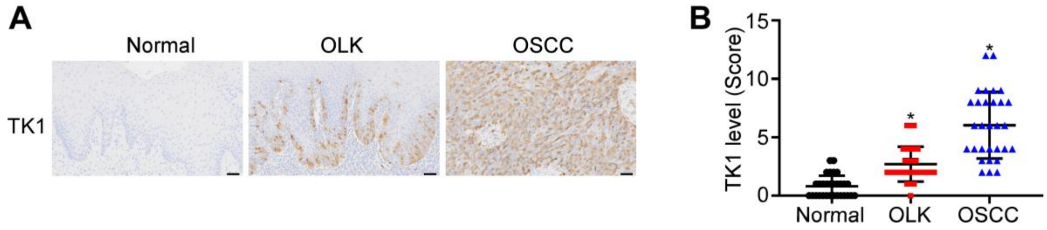

Fig. S7 TK1 increases during OSCC initiation. (A-B) The representative images (×200) (A) and quantification analysis of TK1 (B) protein levels in normal (n = 30), OLK (n = 30), and OSCC (n = 30) tissues were assessed by immunohistochemical staining. Scale bar: 50 μm. Error bars, mean ± SD; $^{*}P < 0.05$.

Figure S8

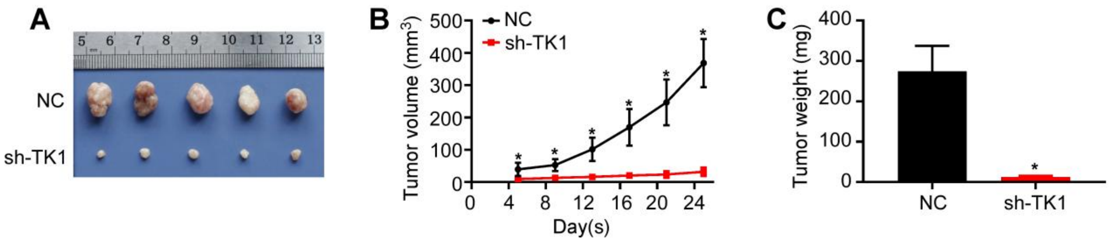

Fig. S8 TK1 promotes OSCC tumor growth *in vivo*. (A-C) The nodules (A), growth curves of tumor volume (B), and tumor weights (C) of CAL27 xenografts transfected with NC (n = 5) and sh-TK1 (n = 5) in BALB/c nude mice. Error bars, mean ± SD; $^{*}P < 0.05$.

Figure S9

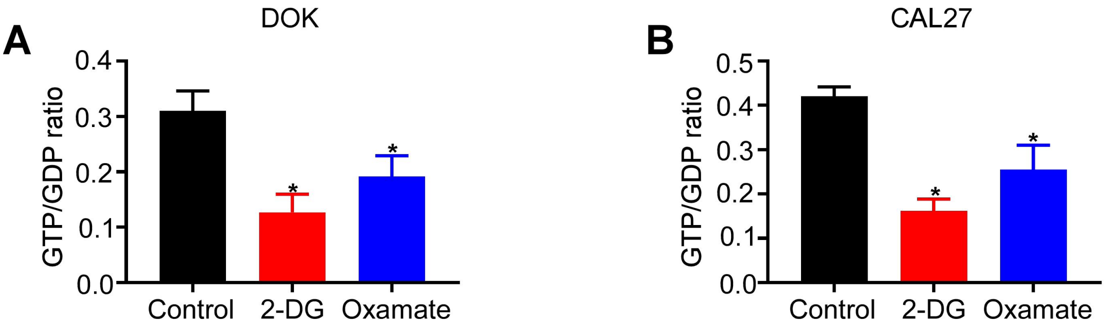

Fig. S9 Suppressive effect of glycolysis inhibitors on the GTP/GDP ratios. DOK (A) and CAL27 (B) cells were treated with the glycolysis inhibitor 2-DG (4 mM) or oxamate (20 mM) for 24 h. GTP/GDP ratios were detected by mass spectrometry. Each experiment was performed in triplicate. Error bars, mean ± SD; $^{*}P < 0.05$.

Table S1 Baseline characteristics of the patients enrolled in IHC and real-time RT-PCR validation

	Normal group	OLK group	OSCC group	
	n (%)	n (%)	n (%)	*P* value[a]
**sex**				
female	9	14	14	0.317
male	21	16	16	
**age**				
<60	20	14	13	0.147
≥ 60	10	16	17	
**OLK site**				
others	25	18		0.084
lateral/ventral tongue	5	12		
**Epithelial dysplasia**				
none		3		
mild		10		
moderate		9		
severe		8		
**OSCC site**				
not tongue			17	
tongue			13	
**OSCC-TNM stage**				

I	9
II	14
III	6
IV	1

[a] χ^2 test.

Table S2 Antibodies information.

Reagent	Company	Catalog No.	Application
Pan Kla	PTM BIO	PTM-1401RM	WB, 1:500
Pan Kla	PTM BIO	PTM-1401RM	IHC, 1:100 (human)
Pan Kla	PTM BIO	PTM-1401RM	IHC, 1:200 (mice)
H3K18la	PTM BIO	PTM-1406RM	WB, 1:500
H3K18la	PTM BIO	PTM-1406RM	IHC, 1:200 (human)
H3K18la	PTM BIO	PTM-1406RM	IHC, 1:200 (mice)
H3K18la	PTM BIO	PTM-1427RM	CUT&Tag, 1:100
Histone H3	Proteintech	17168-1-AP	WB, 1:1000
LDHA	Proteintech	19987-1-AP	WB, 1:5000
β-actin	Proteintech	81115-1-RR	WB, 1:5000
Ki67	Servicebio	GB111141	IHC, 1:1000
TK1	Abcam	ab76495	WB, 1:1000
RhoA	Cell Signaling Technology	2117	WB, 1:1000
β-catenin	Proteintech	51067-2-AP	WB, 1:5000
c-myc	Cell Signaling Technology	5605	WB, 1:1000
P53	Proteintech	10442-1-AP	IHC, 1:1000 (human)

Table S3 Primers used in this study.

Gene	Sequence (5' to 3')
**Real-time RT-PCR primers**	
TK1-F	GGGCAGATCCAGGTGATTCTC
TK1-R	TGTAGCGAGTGTCTTTGGCATA
β-actin-F	CATGTACGTTGCTATCCAGGC
β-actin-R	CTCCTTAATGTCACGCACGAT
**siRNA sequences**	
siRNA-NC-F	UUCUCCGAACGUGUCACGUTT
siRNA-NC-R	ACGUGACACGUUCGGAGAATT
siLDHA-F	ACCAUGAUUAAGGGUCUUUTT
siLDHA-R	AAAGACCCUUAAUCAUGGUGG
**ChIP-qPCR primers**	
TK1-F	TTAATGCAGCTCATTGCGCC
TK1-R	GGGAACCAGGGGCTTACTG
