# Supplementary material for: Histone lactylation-driven feedback loop modulates pyrimidine metabolism to promote oral carcinogenesis
Source: Cell Death Dis. 2026 Mar 19;17(1):316. doi: 10.1038/s41419-026-08580-w (PMC13039119; doi:10.1038/s41419-026-08580-w)
Supplement: Supplementary file 2 — Original Western blots [file 41419_2026_8580_MOESM2_ESM.pptx]

## Slide 1
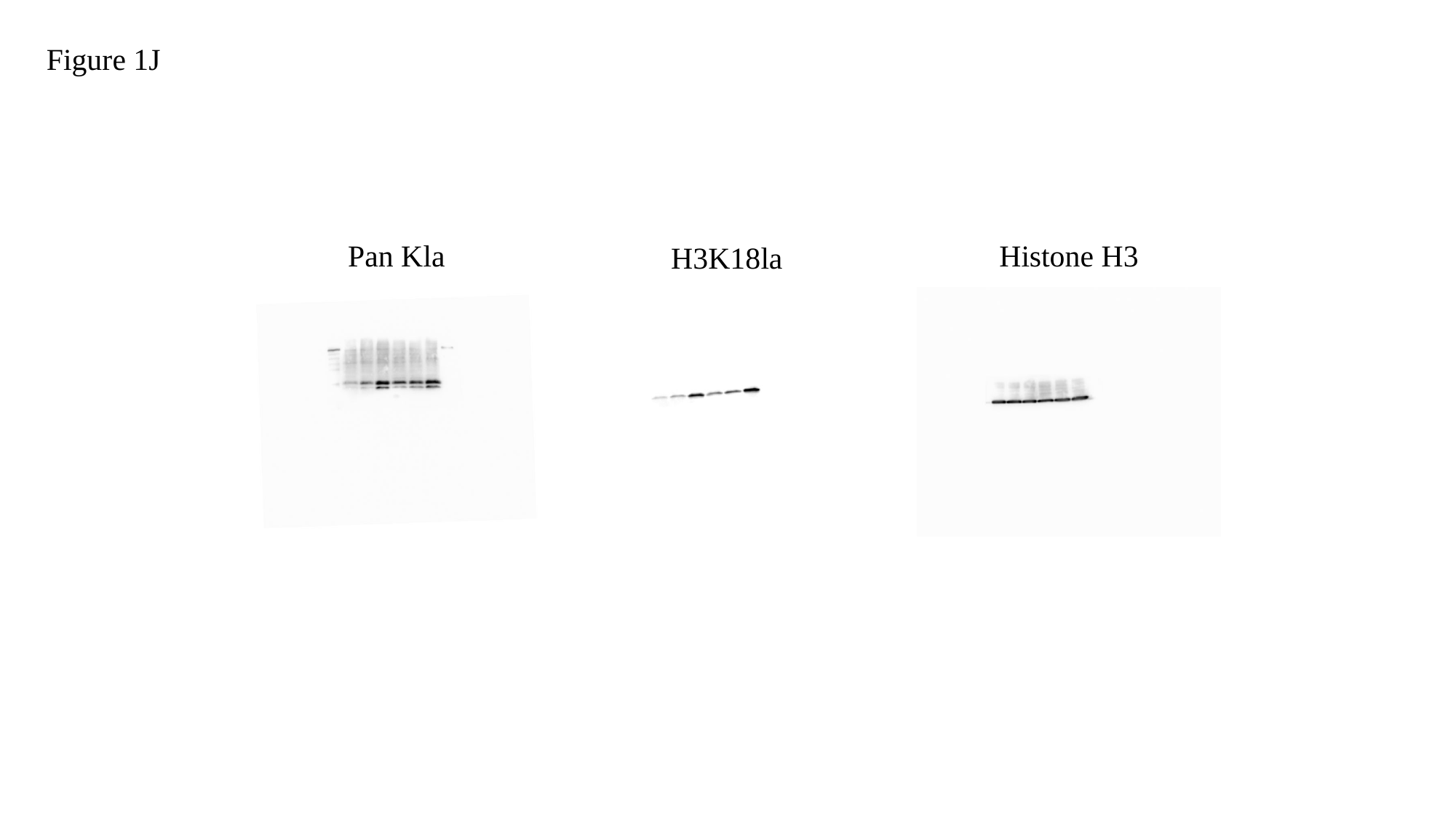

Figure 1J
Pan Kla
Histone H3
H3K18la

## Slide 2
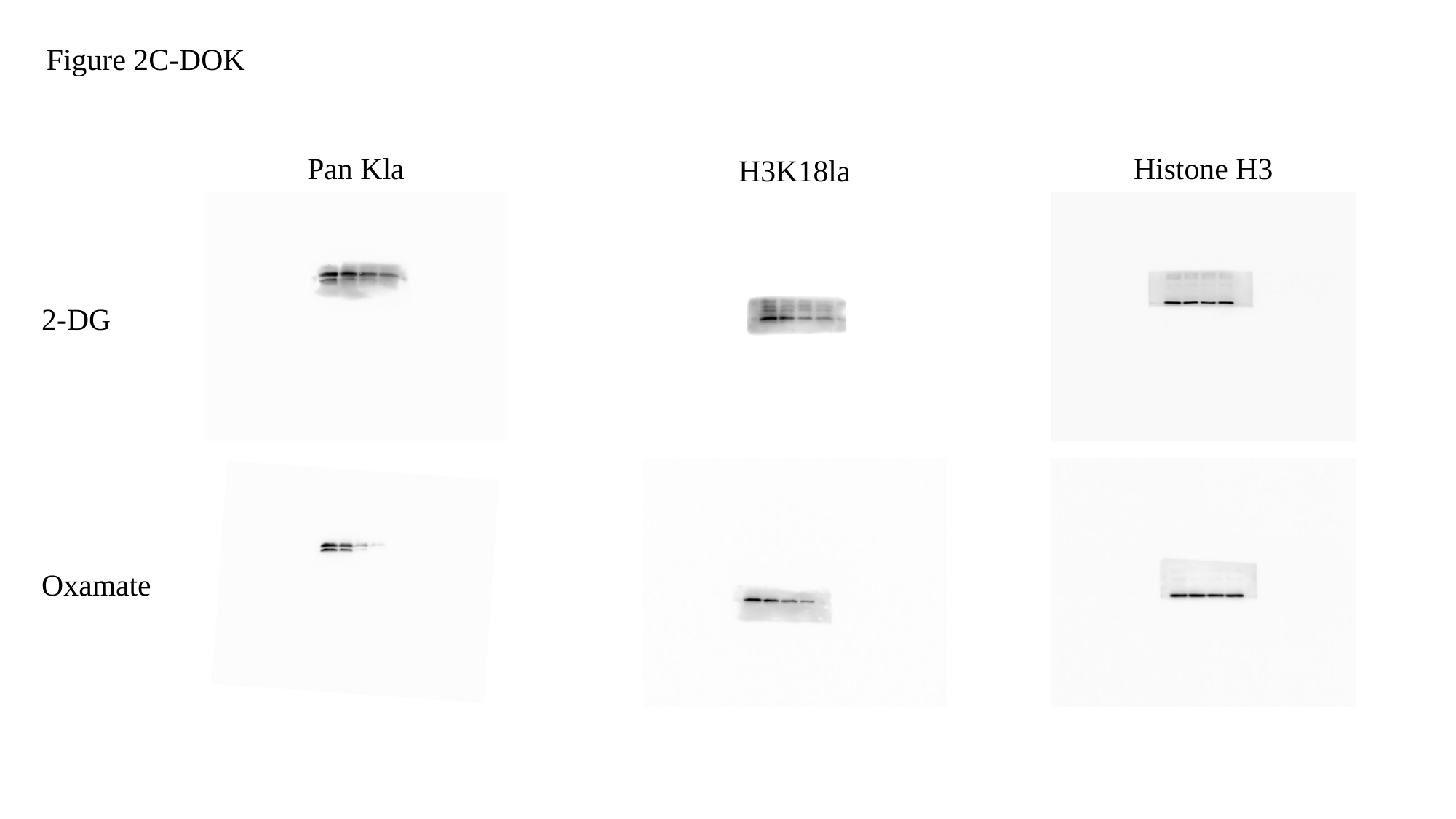

Figure 2C-DOK
Pan Kla
Histone H3
H3K18la
2-DG
Oxamate

## Slide 3
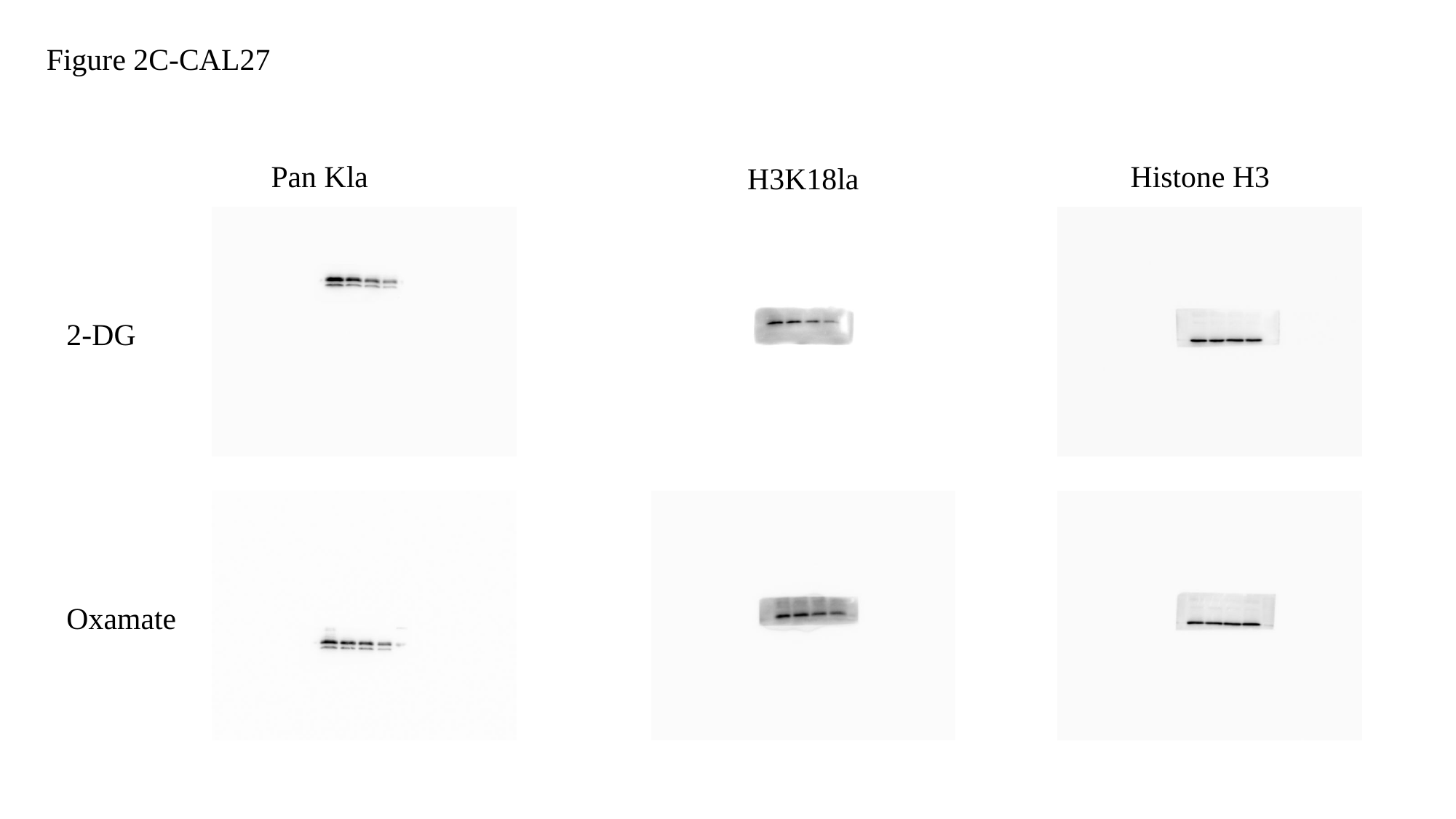

Figure 2C-CAL27
Pan Kla
Histone H3
H3K18la
2-DG
Oxamate

## Slide 4
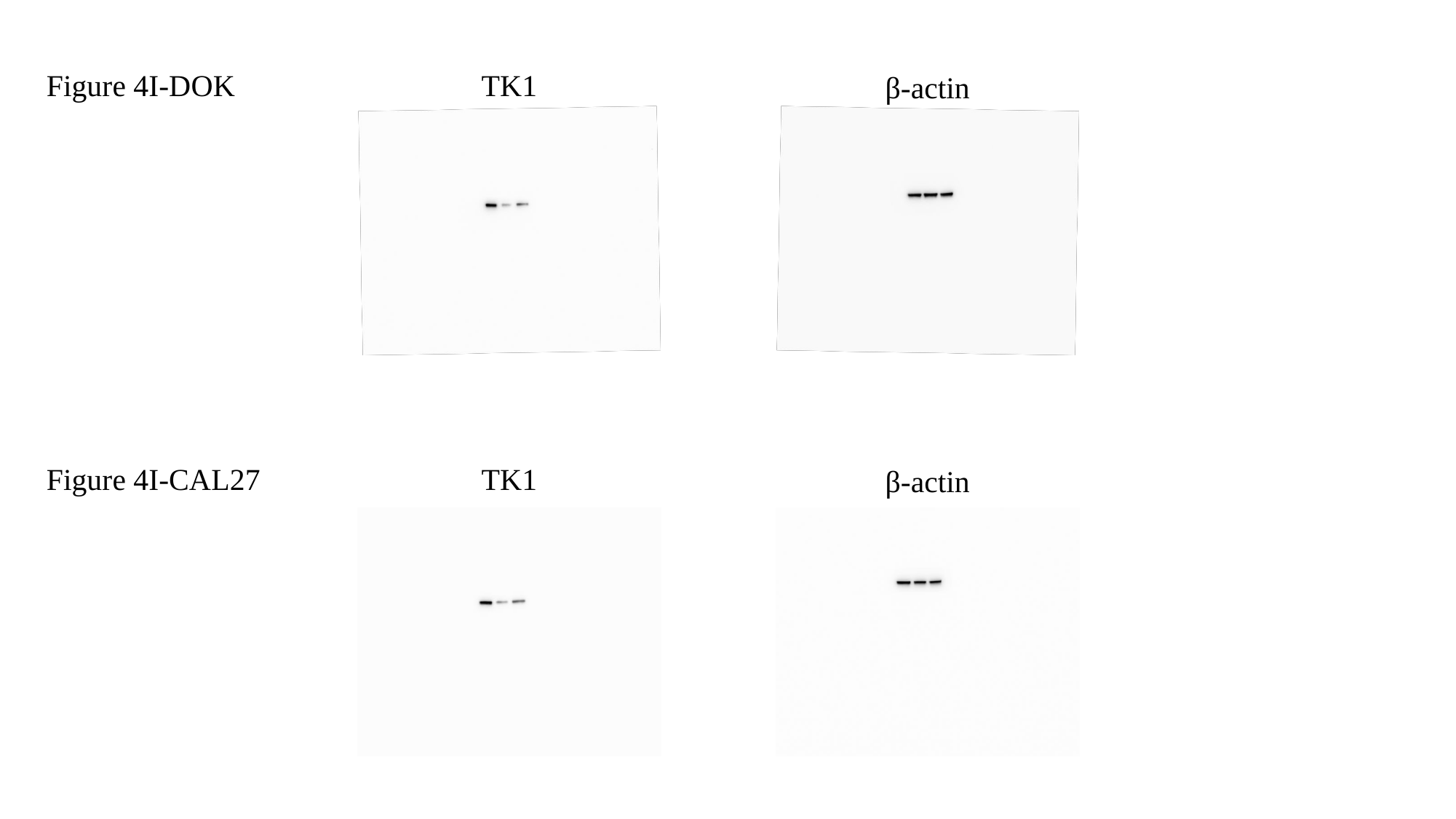

Figure 4I-DOK
TK1
β-actin
Figure 4I-CAL27
TK1
β-actin

## Slide 5
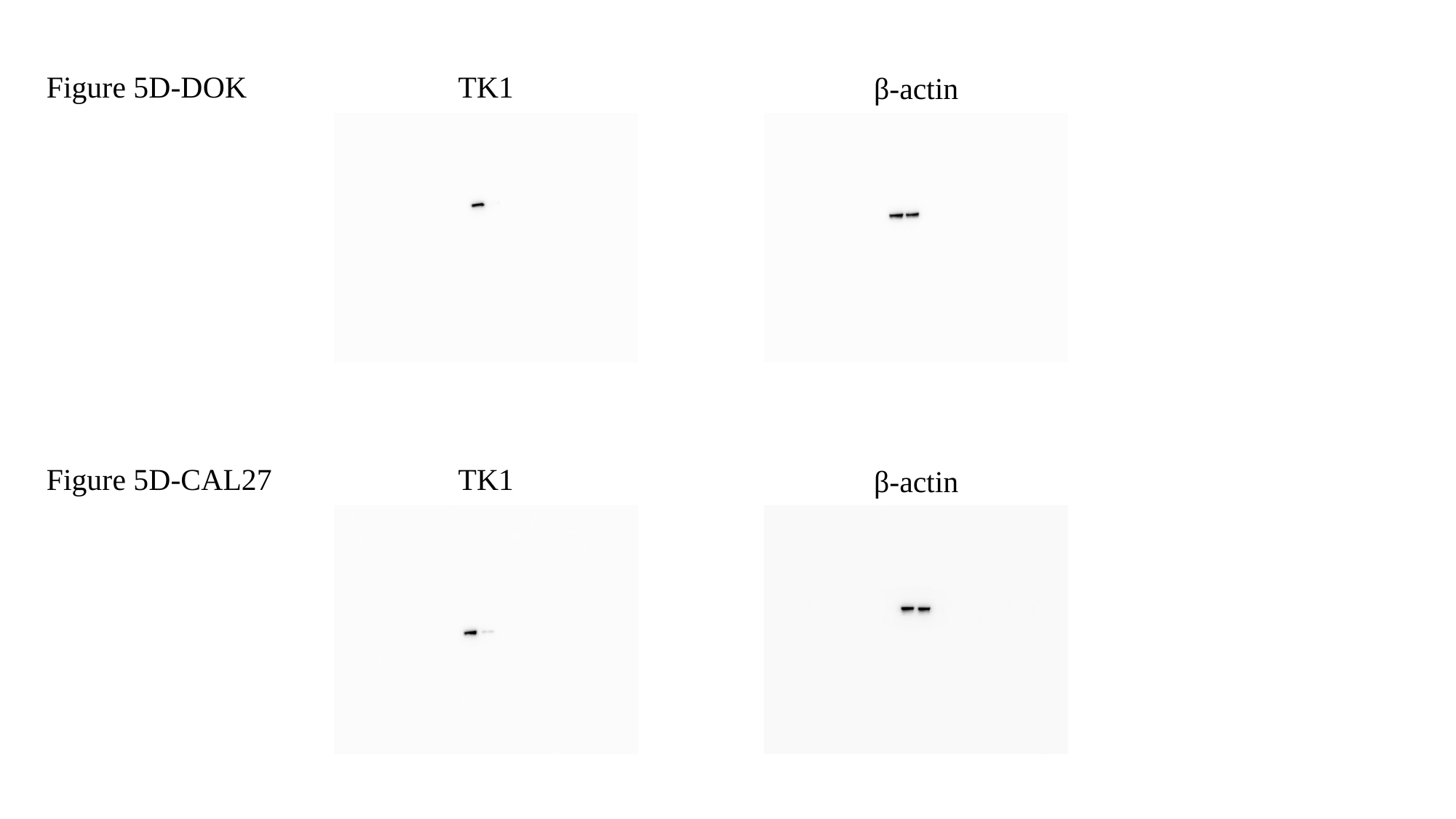

Figure 5D-DOK
TK1
β-actin
Figure 5D-CAL27
TK1
β-actin

## Slide 6
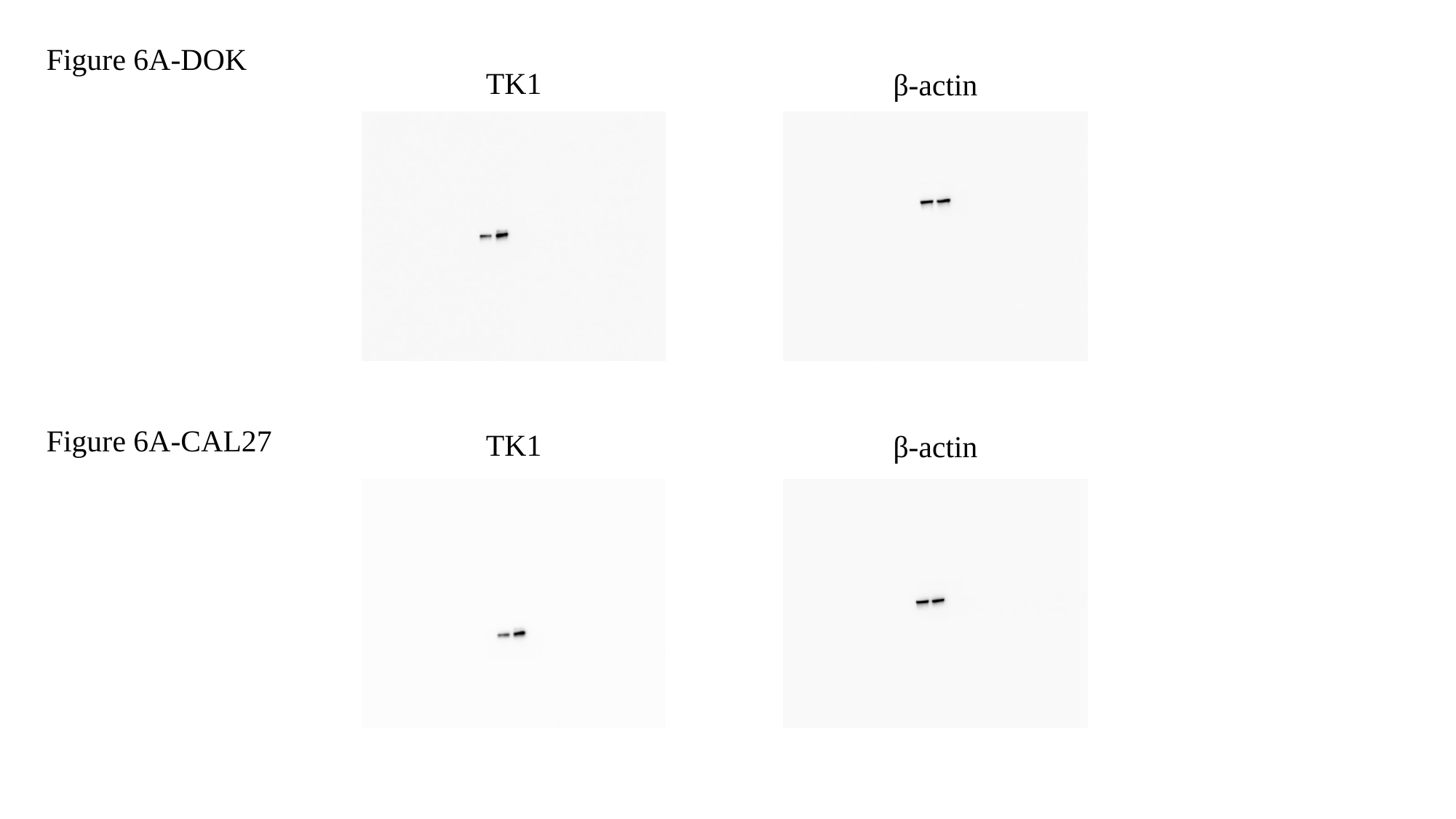

Figure 6A-DOK
TK1
β-actin
Figure 6A-CAL27
TK1
β-actin

## Slide 7
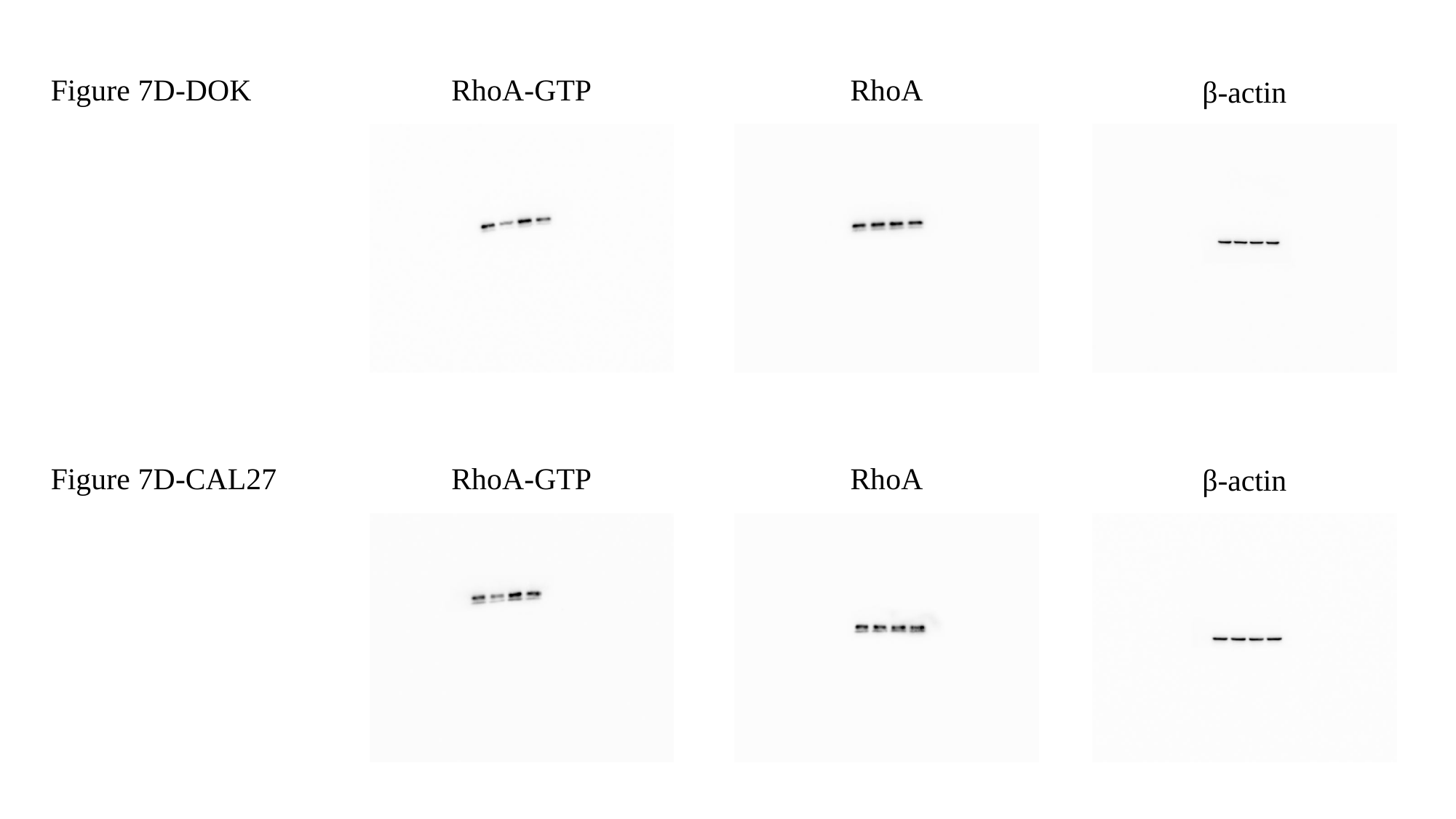

Figure 7D-DOK
RhoA-GTP
RhoA
β-actin
Figure 7D-CAL27
RhoA-GTP
RhoA
β-actin

## Slide 8
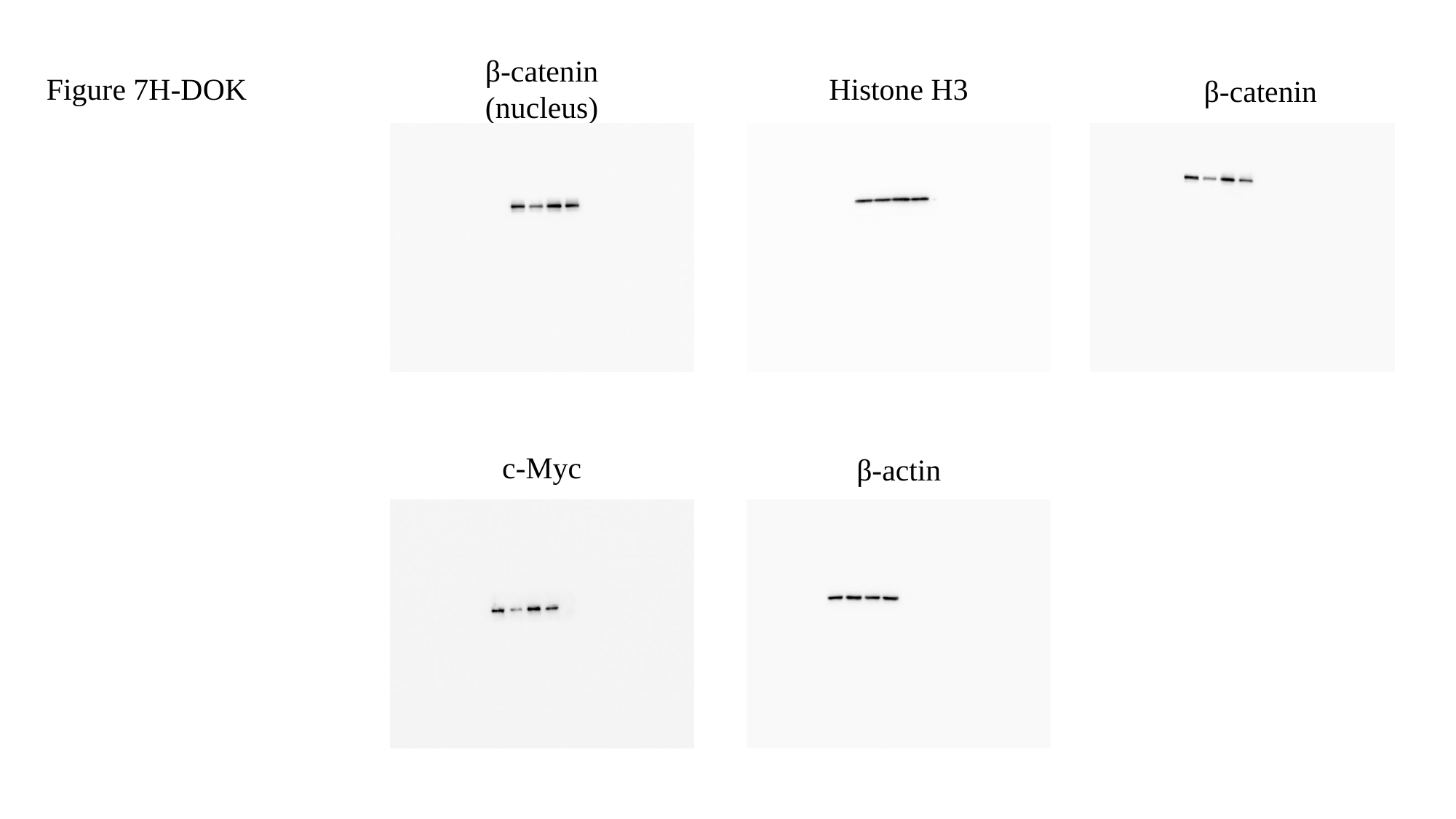

β-catenin
(nucleus)
Figure 7H-DOK
Histone H3
β-catenin
c-Myc
β-actin

## Slide 9
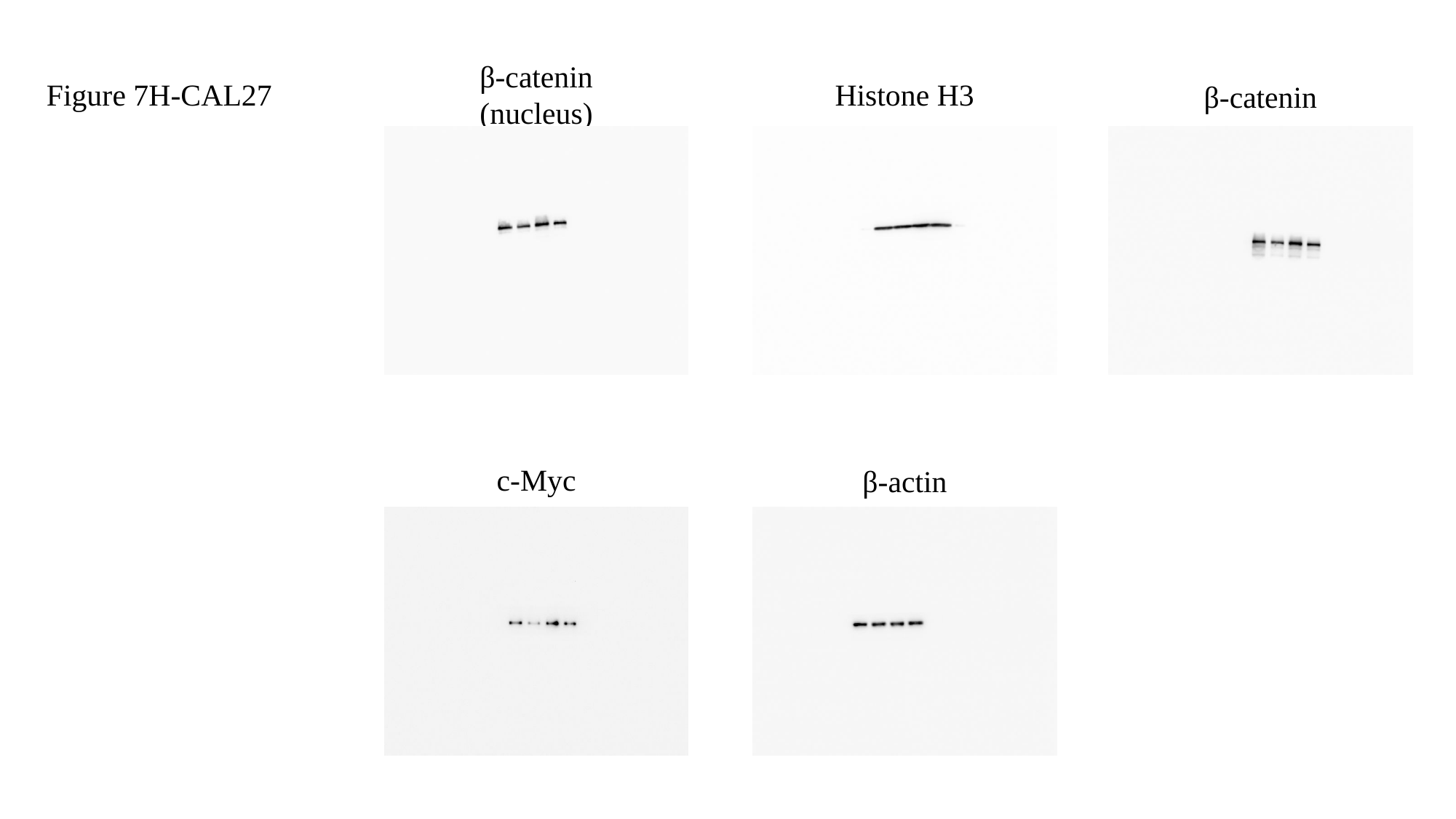

β-catenin
(nucleus)
Figure 7H-CAL27
Histone H3
β-catenin
c-Myc
β-actin

## Slide 10
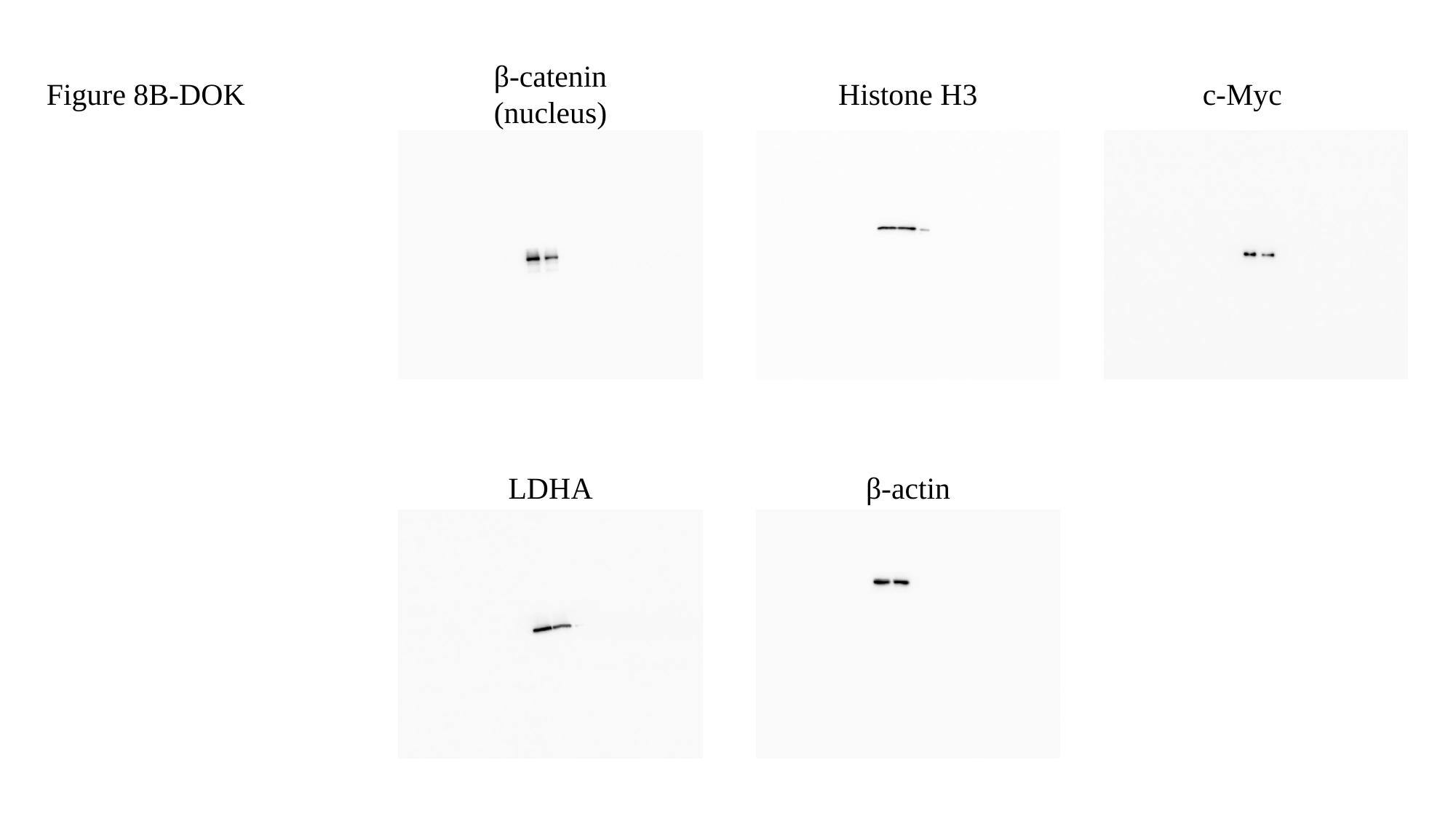

β-catenin
(nucleus)
Figure 8B-DOK
Histone H3
c-Myc
LDHA
β-actin

## Slide 11
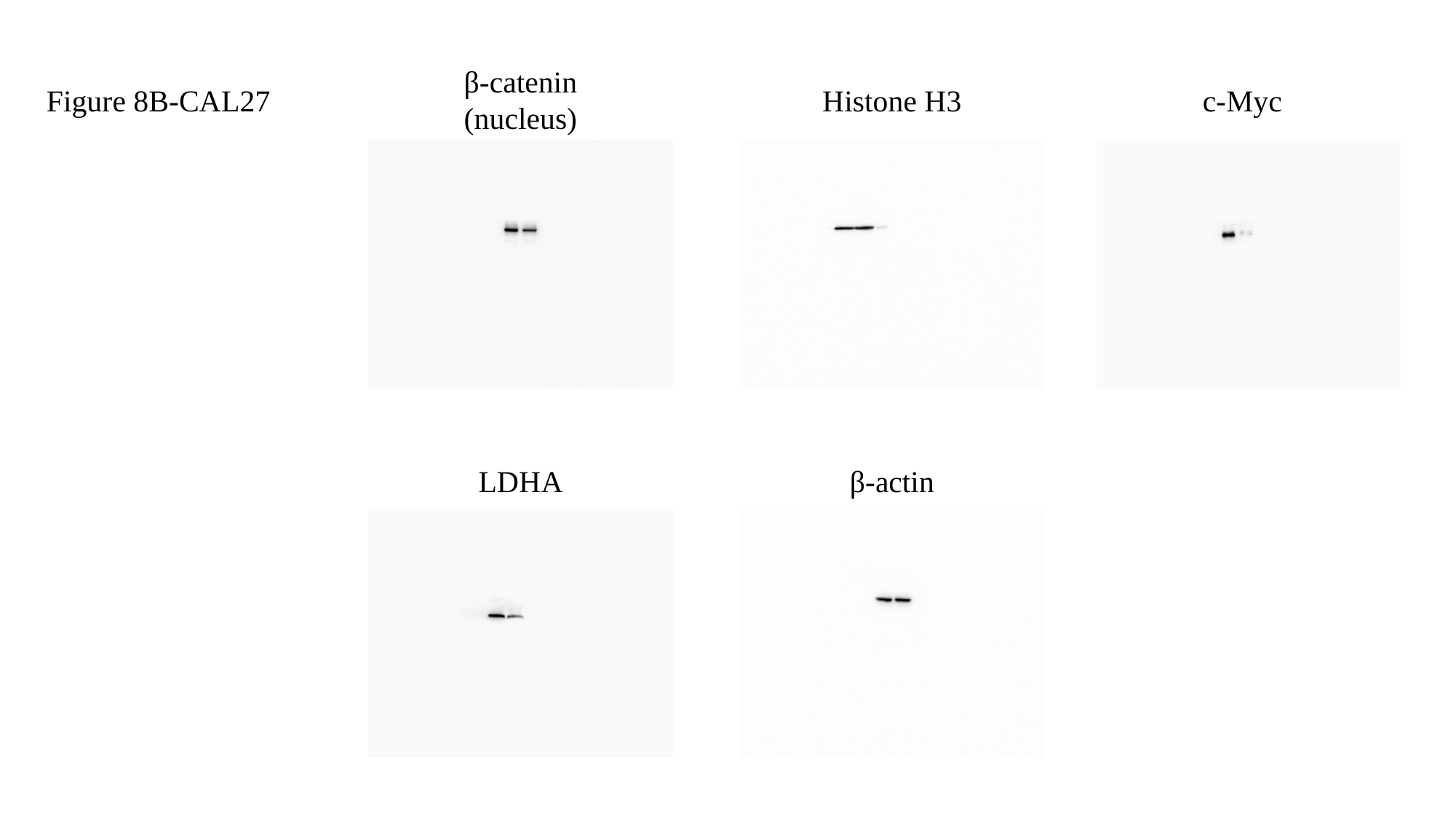

β-catenin
(nucleus)
Figure 8B-CAL27
Histone H3
c-Myc
LDHA
β-actin

## Slide 12
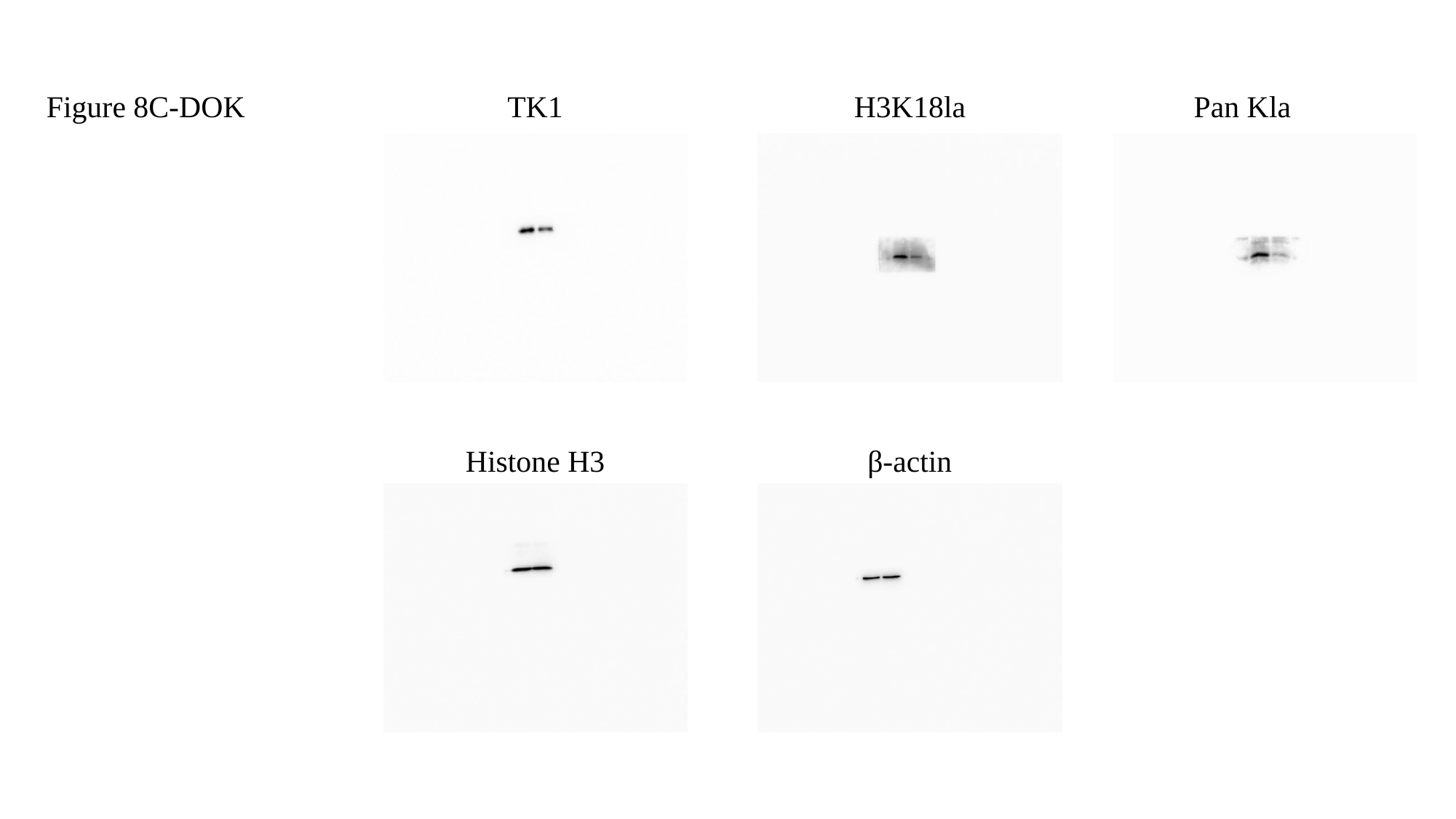

Figure 8C-DOK
TK1
H3K18la
Pan Kla
Histone H3
β-actin

## Slide 13
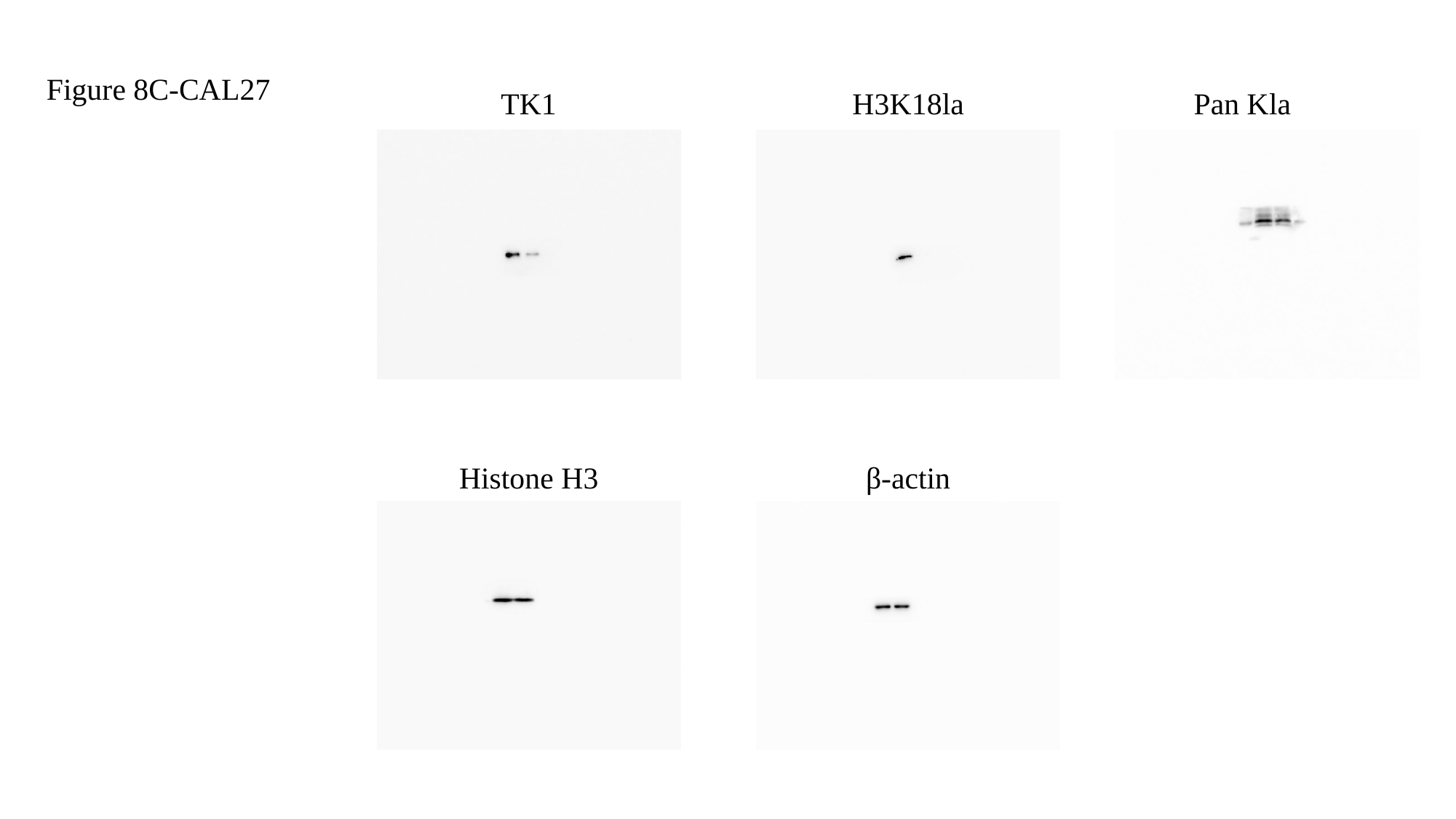

Figure 8C-CAL27
TK1
H3K18la
Pan Kla
Histone H3
β-actin

## Slide 14
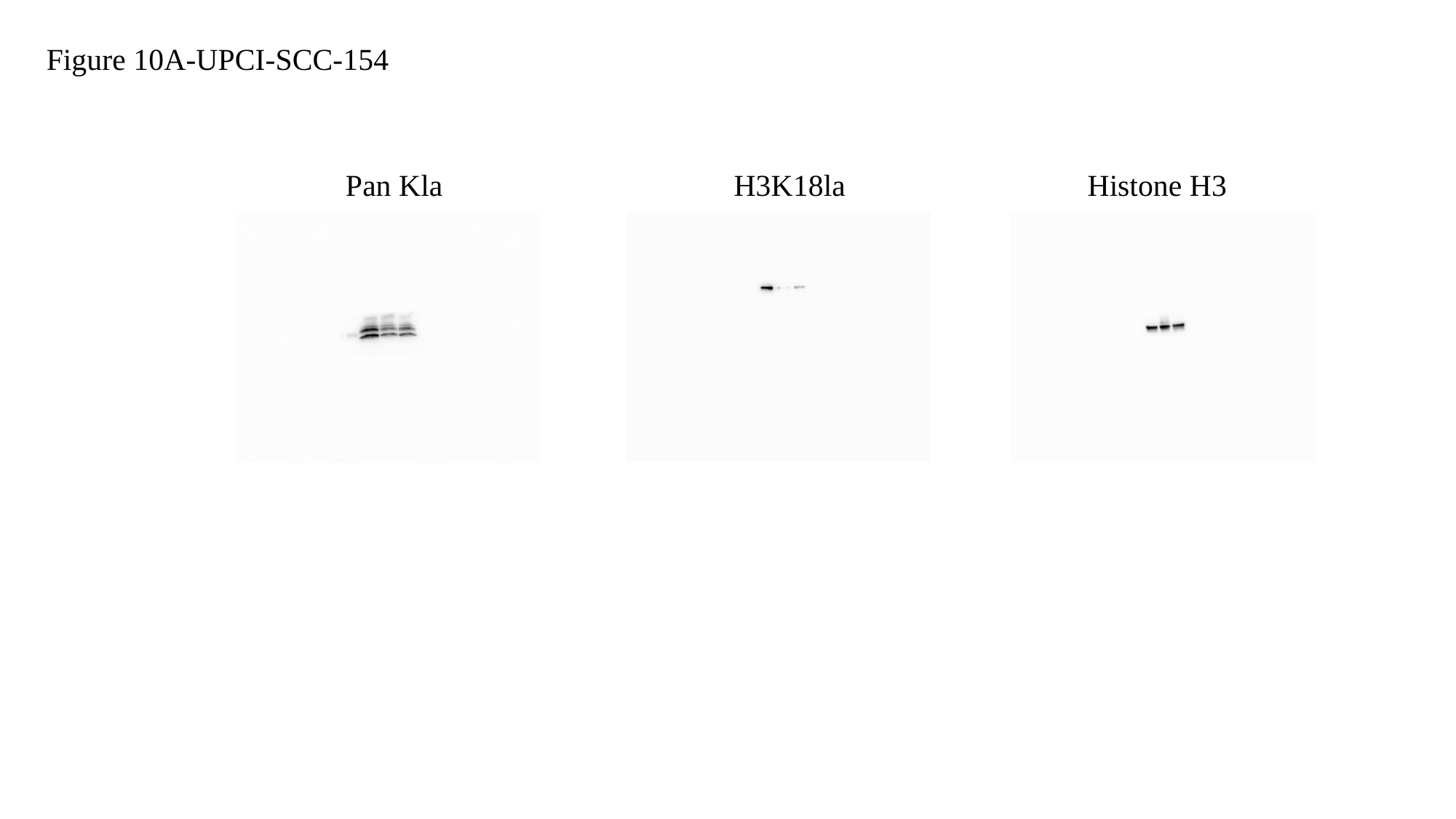

Figure 10A-UPCI-SCC-154
Pan Kla
H3K18la
Histone H3

## Slide 15
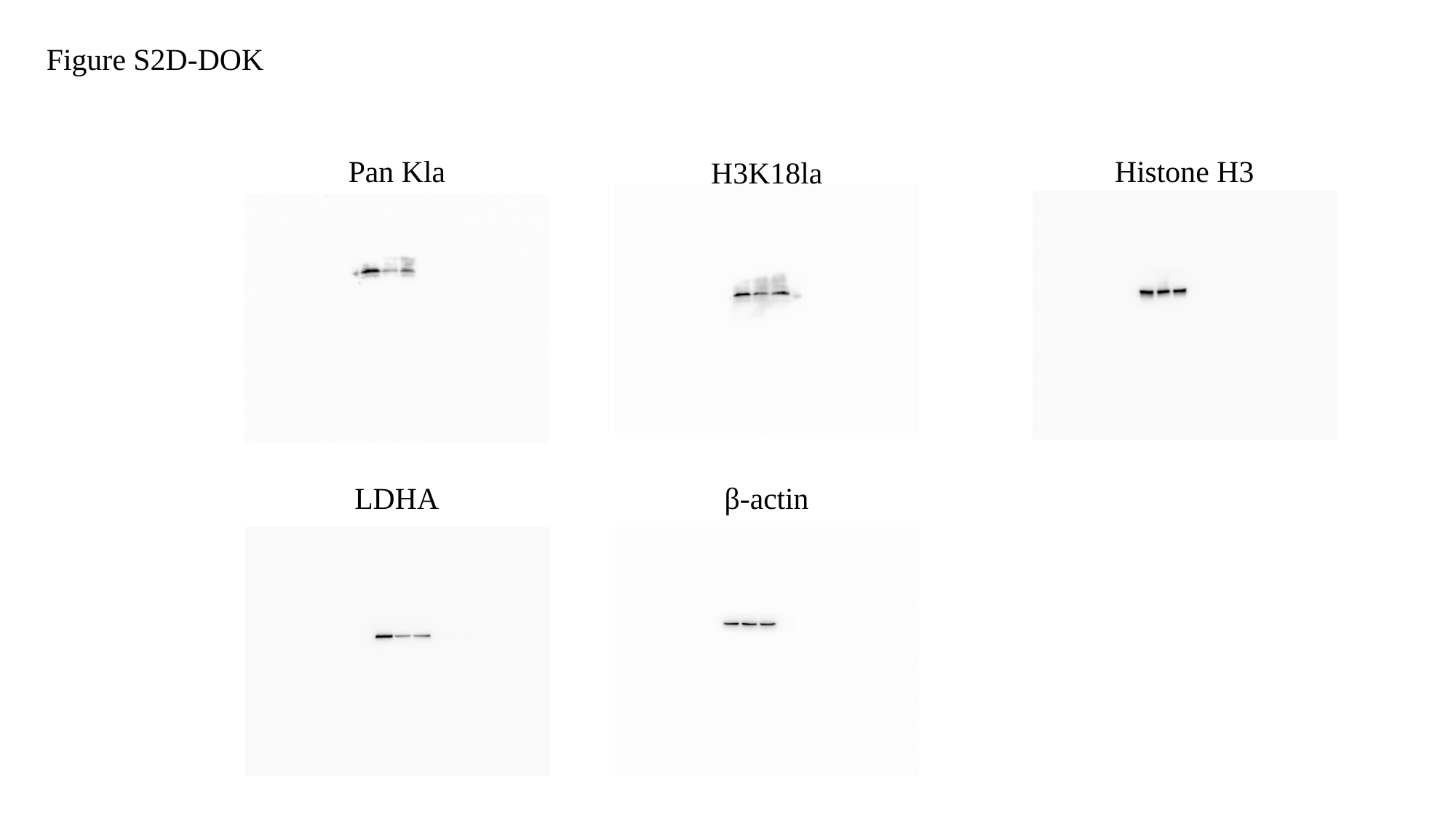

Figure S2D-DOK
Pan Kla
Histone H3
H3K18la
LDHA
β-actin

## Slide 16
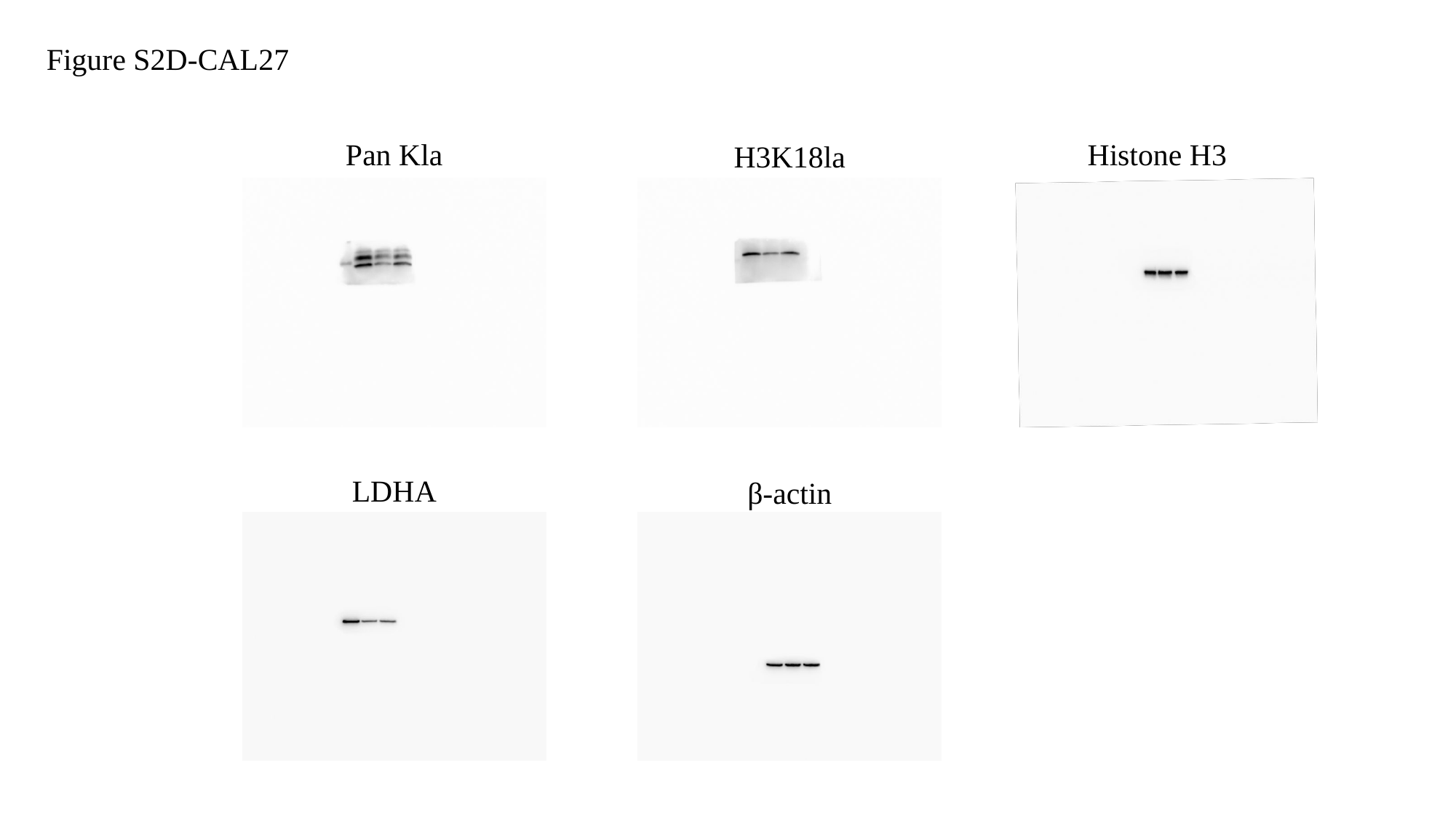

Figure S2D-CAL27
Pan Kla
Histone H3
H3K18la
LDHA
β-actin
